# MicroRNA-22 Can Reduce Parathymosin Expression in Transdifferentiated Hepatocytes

**DOI:** 10.1371/journal.pone.0034116

**Published:** 2012-04-06

**Authors:** Hung-Lin Chen, Jyun-Yuan Huang, Chun-Ming Chen, Tien-Hua Chu, Chiaho Shih

**Affiliations:** 1 Institute of Biomedical Sciences, Academia Sinica, Taipei, Taiwan; 2 Graduate Institute of Life Sciences, National Defense Medical Center, Taipei, Taiwan; Yonsei University, Republic of Korea

## Abstract

Pancreatic acinar cells AR42J-B13 can transdifferentiate into hepatocyte-like cells permissive for efficient hepatitis B virus (HBV) replication. Here, we profiled miRNAs differentially expressed in AR42J-B13 cells before and after transdifferentiation to hepatocytes, using chip-based microarray. Significant increase of miRNA expression, including miR-21, miR-22, and miR-122a, was confirmed by stem-loop real-time PCR and Northern blot analyses. In contrast, miR-93, miR-130b, and a number of other miRNAs, were significantly reduced after transdifferentiation. To investigate the potential significance of miR-22 in hepatocytes, we generated cell lines stably expressing miR-22. By 2D-DIGE, LC-MS/MS, and Western blot analyses, we identified several potential target genes of miR-22, including parathymosin. In transdifferentiated hepatocytes, miR-22 can inhibit both mRNA and protein expression of parathymosin, probably through a direct and an indirect mechanism. We tested two computer predicted miR-22 target sites at the 3′ UTR of parathymosin, by the 3′ UTR reporter gene assay. Treatment with anti-miR-22 resulted in significant elevation of the reporter activity. In addition, we observed an in vivo inverse correlation between miR-22 and parathymosin mRNA in their tissue distribution in a rat model. The phenomenon that miR-22 can reduce parathymosin protein was also observed in human hepatoma cell lines Huh7 and HepG2. So far, we detected no major effect on several transdifferentiation markers when AR42J-B13 cells were transfected with miR-22, or anti-miR-22, or a parathymosin expression vector, with or without dexamethasone treatment. Therefore, miR-22 appears to be neither necessary nor sufficient for transdifferentiation. We discussed the possibility that altered expression of some other microRNAs could induce cell cycle arrest leading to transdifferentiation.

## Introduction

Transdifferentiation from one differentiated cell type to another can occur *in vivo* and *in vitro*. For example, hepatic foci can be found within pancreas in experimental animal models and human patients [1], [2]. AR42J-B13 is a rat pancreatic acinar cell line, which can transdifferentiate into hepatocytes by treatment with dexamethasone (Dex) and oncostatin M (OSM) [3]. Transdifferentiated hepatocytes of AR42J-B13 cells expressed several liver-specific markers and transcription factors [3], [4]. Interestingly, these rodent hepatocyte-like cells can support very efficient cross-species replication of human hepatitis B virus – a hepatotropic virus [3]–[5]. Recently, microRNAs (miRs) were found to regulate many important biological processes including tumorigenesis and development [6], [7]. MicroRNAs can inhibit gene expression through base-pairing of their seed sequences to the 3′UTR of target genes, leading to translational suppression or mRNA degradation. However, whether miRNAs could play a role in hepatic transdifferentiation remains to be investigated.

The expression of tissue specific miRNAs is often regulated at the transcription level by tissue specific transcription factors (for examples, miR-122a by HNF4, miR-192 and miR-194 by HNF1 [8], [9]. Furthermore, liver specific miR-122a has been reported to regulate many genes involved in cholesterol and fatty-acid metabolism [9]–[11]. Therefore, to identify differentially expressed miRNAs is an important approach to a better understanding of the roles of miRNAs during transdifferentiation. Here, we profiled the expression levels of miRNAs before and after transdifferentiation of AR42J-B13 cells by microarray analysis. The results of microarray analysis were confirmed by stem-loop real-time PCR and Northern blot analyses. Among a number of miRNAs with significantly altered expression levels, we focused on the less well studied miR-22 in hepatocytes.

MicroRNA-22 is a highly evolutionarily conserved microRNA present in various primary tissues and cell lines [12]. Recently, miR-22 was found to be involved in various signaling pathways, including estrogen, PTEN/AKT, and c-Myc [13]–[15]. Ectopic overexpression of miR-22 inhibits cell proliferation and induced G1 cell cycle arrest, while a lower expression level of miR-22 correlated with a higher degree of tumorigenicity in human hepatocellular carcinoma patients [15]–[17]. Previous studies reported the potential roles of miR-22 in heart and muscle [14], [16]. In this study, we took an approach of proteomic analysis to identify direct or indirect targets of miR-22 in transdifferentiated hepatocytes. One of the potential targets of miR-22 that we identified here is parathymosin.

Parathymosin is a small nuclear protein that can physically interact with glucocorticoid receptors (GR) and histone H1, respectively, and can serve as a coactivator of GR [18], [19]. We observed here the reduction of parathymosin mRNA and protein levels in AR42J-B13 cells treated with Dex/OSM or with a miR-22 expression vector. We also demonstrated here that miR-22 could reduce the expression of parathymosin by binding to two predicted sites at the 3′ UTR of parathymosin gene. Treatment of antagomiR-22 (anti-miR22) resulted in an increased expression of the reporter containing the 3′UTR of parathymosin. In addition, anti-miR treatment can result in increased expression of parathymosin mRNA or protein in rat AR42J-B13 cells and human HepG2 and Huh7 cells. Taken together, we identified miR-22 as a microRNA species highly elevated in transdifferentiated hepatocytes. One of the potential targets of miR-22 is parathymosin. Further studies of the relationships among miRNAs and their targets should contribute to our understanding of hepatic transdifferentiation in AR42J-B13 cells.

## Results

### MiRNA expression profiles during AR42J-B13 hepatic transdifferentiation

To examine the expression profiles of miRNAs during Dex/OSM induced AR42J-B13 transdifferentiation, we used chip-based miRNA microarray analysis. By comparing the miRNA expression profiles of AR42J-B13 cells before and after transdifferentiation, we observed four major patterns of miRNA expression: 1) the majority of miRNAs were expressed at low levels, regardless of the transdifferentiation status (data not shown). 2) Some miRNAs, including let-7 family (let-a, -b and -c), miR-16, miR-23b, miR-26, miR-31 and miR-375, were always highly expressed either before or after transdifferentiation (data not shown). 3) We identified 29 down-regulated miRNAs (Table 1). 4) We also identified 13 up-regulated miRNAs with fold changes greater than 2 in two independent experiments (Table 2).

**Table 1:** Decreased miRNA expression profile of AR42J-B13 cells after transdifferentiation by miRNA microarray*.

miRNA	Fold of decrease	
	Exp1	Exp2
miR-181b	7.14	5.00
miR-106a	6.25	5.56
miR-130b	6.25	5.56
miR-17-5p	6.25	5.88
miR-93	5.88	5.88
miR-106b	5.56	5.00
miR-15b	5.56	3.70
miR-25	5.56	3.70
miR-335	5.56	4.00
miR-138	5.00	3.03
miR-181a	4.55	4.17
miR-18a	4.35	2.86
miR-181d	4.00	2.70
miR-19b	3.70	3.85
miR-20a	3.70	2.78
miR-7i	3.24	2.28
miR-342	3.23	3.03
miR-130a	3.13	2.63
miR-20b	3.13	2.56
miR-27b	3.13	3.03
miR-31	3.13	2.33
miR-672	3.03	2.50
miR-375	2.94	3.13
let-7d	2.86	2.38
miR-16	2.70	2.94
miR-92	2.56	2.78
miR-99a	2.44	2.78
miR-99b	2.22	2.44
miR-23b	2.17	2.38

* MicroRNA microarray was used to profile differential miRNA expression during AR42J-B13 transdifferentiation. The fold of decrease represented the reduction of miRNAs after transdifferentiation. Results from two independent microarray analysis with fold changes greater than 2 were shown in the Table.

**Table 2:** Increased miRNA expression profile of AR42J-B13 cells after transdifferentiation by miRNA microarray*.

miRNA	Fold of increase	
	Exp1	Exp2
miR-122a	76.92	74.07
miR-21	23.42	22.37
miR-22	16.16	11.35
miR-422b	4.69	2.92
miR-30b	3.47	2.48
miR-29a	3.4	3.13
miR-182	3.28	3.29
miR-29c	3.1	2.34
miR-101b	2.79	2.04
miR-192	2.39	2.26
miR-29b	2.34	2.01
miR-30d	2.33	2.08
miR-194	2.24	2.10

* MicroRNA microarray was used to profile differential miRNA expression during AR42J-B13 transdifferentiation. The fold of increase represented the enrichment of miRNAs after transdifferentiation. Results from two independent microarray analysis with fold changes greater than 2 were shown in the Table.

**Figure 1:**
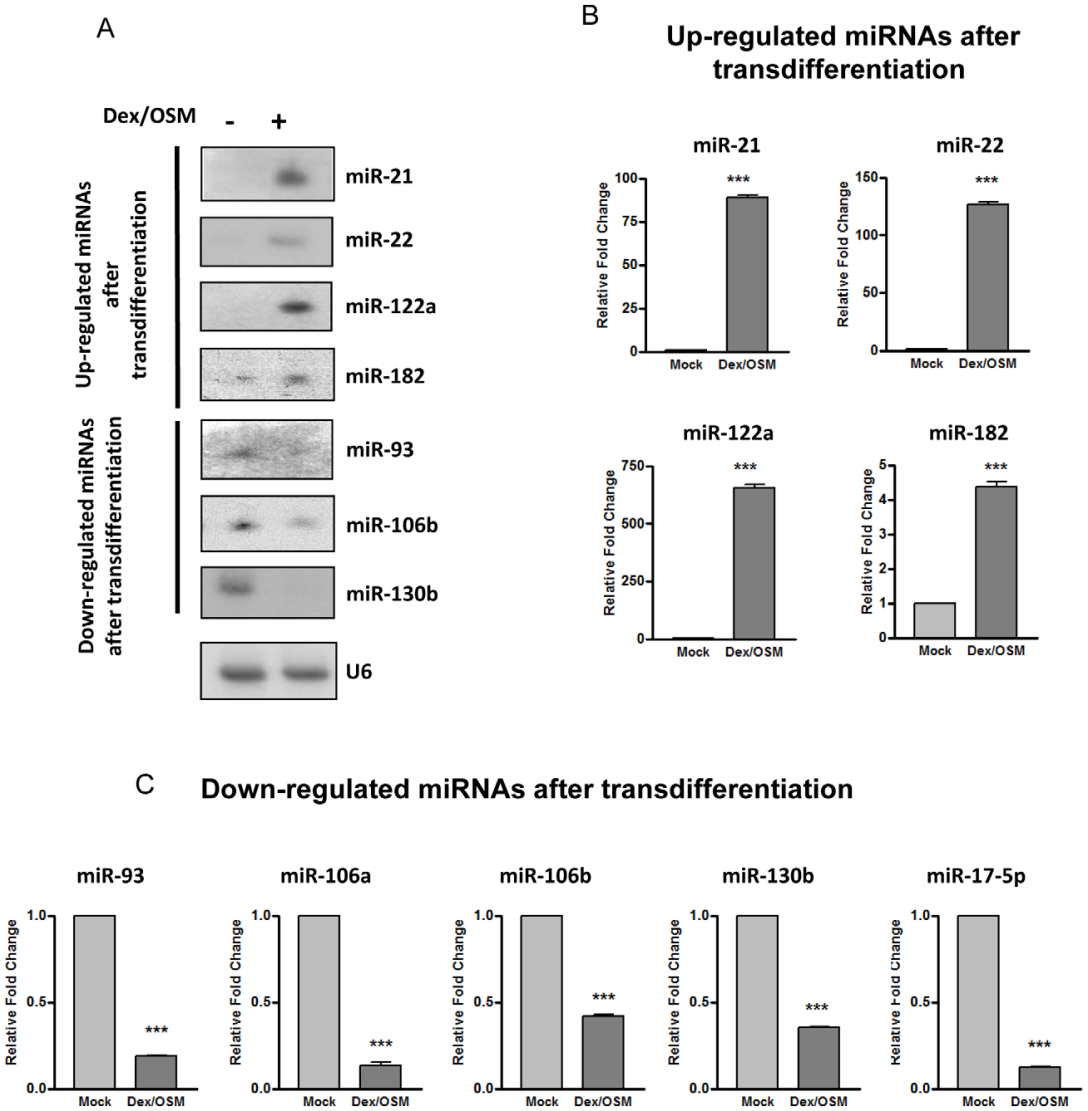
Northern blot and stem-loop real-time PCR analysis of differentially expressed miRNAs before and after transdifferentiation of AR42J-B13 cells. The differentially expressed miRNAs identified by miRNA microarray were confirmed by miRNA Northern blot (A) and stem-loop real-time PCR analysis (B and C). Total RNA extracted from Dex/OSM treated AR42J-B13 cells (7 Days) and mock controls were used for Northern blot analysis using antisense probes against down-regulated miRNAs (miR-93, miR-106b and miR-130b) and up-regulated miRNAs (miR-21, miR-22, miR-122a and miR-182). The relative fold changes of stem–loop real-time PCR were normalized to AR42J-B13 cells before transdifferentiation and U6 was used as a loading control. (*** P<0.001).

Among the 13 up-regulated miRNAs, miR-122a, miR-21, miR-22 were the most enriched in transdifferentiated hepatocytes with a fold change of ∼70, ∼20 and ∼10, respectively (Table 2). Relative to the up-regulated miRNAs (increased by 2 to 70 fold), the magnitude of decrease in the down-regulated miRNAs was modest (less than 7 fold). The results in Table 1 and 2 were further validated by Northern blot (Fig. 1A and Figure S1) and stem-loop real-time PCR analysis (Fig. 1B and 1C). In general, the fold change of expression of abundant miRNAs, such as miR-122a, miR-21, and miR-22, was much more pronounced in the stem-loop PCR assay than the microarray assay due to the sensitivity and linear range of the PCR assay.

### Transdifferentiated AR42J-B13 cells are hepatocyte-like based on the clustering analysis of miRNA expression profiles between hepatocytes and non-hepatocytes

**Figure 2:**
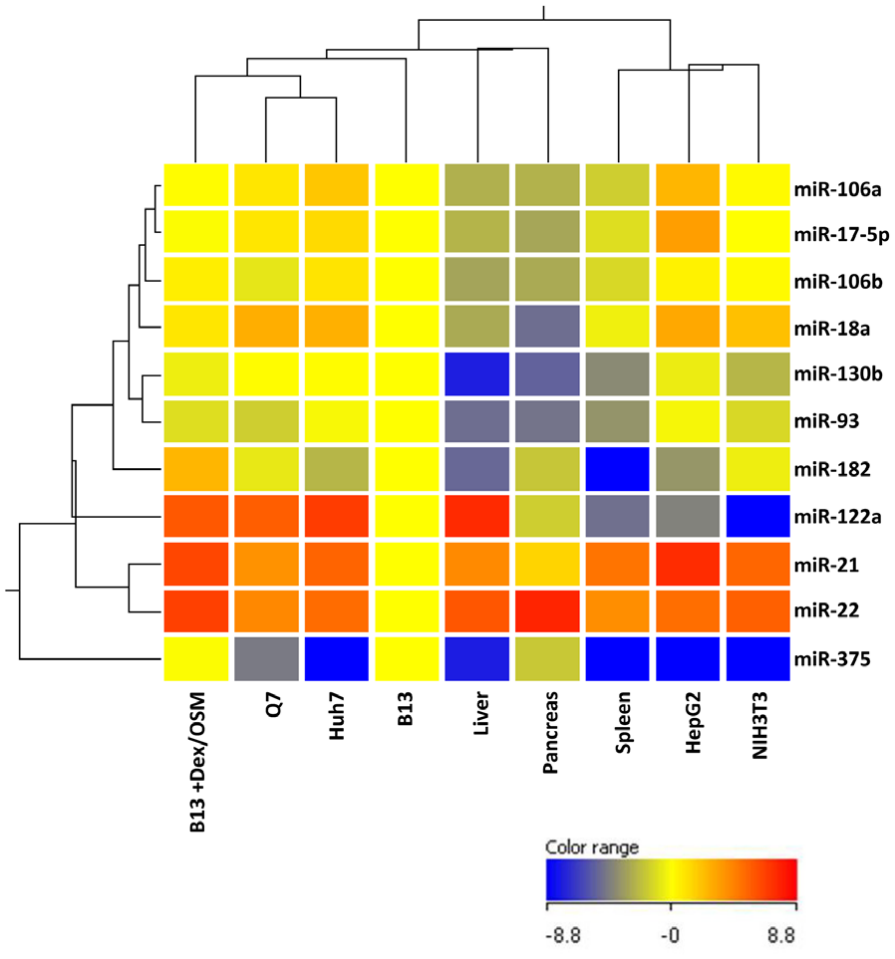
The miRNA expression profile of transdifferentiated AR42J-B13 cells is most closely related to those of hepatoma cell lines by clustering analysis. The expression levels of miRNAs of various hepatoma cell lines (HepG2, Huh7 and Q7 cells), primary tissues (rat spleen, pancreas and liver), and NIH3T3 cells, were measured by stem-loop real-time PCR analysis. Clustering analysis using Genespring V11.0 software was performed by the normalization of the expression level from each sample relative to that of AR42J-B13 cells. Both up-regulated miRNAs (miR-21, miR-22, miR-122a and miR-182) and down-regulated miRNAs (miR-17-5p, miR-18a, miR-93, miR-106a, miR-106b, miR-130b and miR-375) were chosen as a parameter for comparison.

We investigated further by stem-loop real-time PCR analysis that these three most abundant miRNA species in transdifferentiated hepatocyte-like cells were indeed also highly expressed in rat liver *in vivo* as well as hepatoma cell lines *in vitro*, including Huh7 and Morris hepatoma 7777 (Q7 cells in [5]) (data not shown). Such a miRNA expression signature has been well documented in cancer profiling and classification [20]. Using these hepatocyte and non-hepatocyte cell lines and primary tissues, we performed unsupervised clustering analysis by selecting 7 down-regulated miRNAs (miR-17-5p, miR-18a, miR-93, miR-106a, miR-106b, miR-130b and miR-375) and 4 up-regulated miRNAs (miR-21, miR-22, miR-122a and miR-182). The results revealed that the transdifferentiated AR42J-B13 cells exhibited a miRNA signature profile most closely related to hepatoma cell lines, such as Q7 and Huh7 (Fig. 2). In part because of the very low level expression of miR-122a, human hepatoblastoma cell line HepG2 was, to our surprise, clustered into non-hepatocytes.

### Identification of miR-22 target by 2D-DIGE and LC/MS/MS

**Figure 3:**
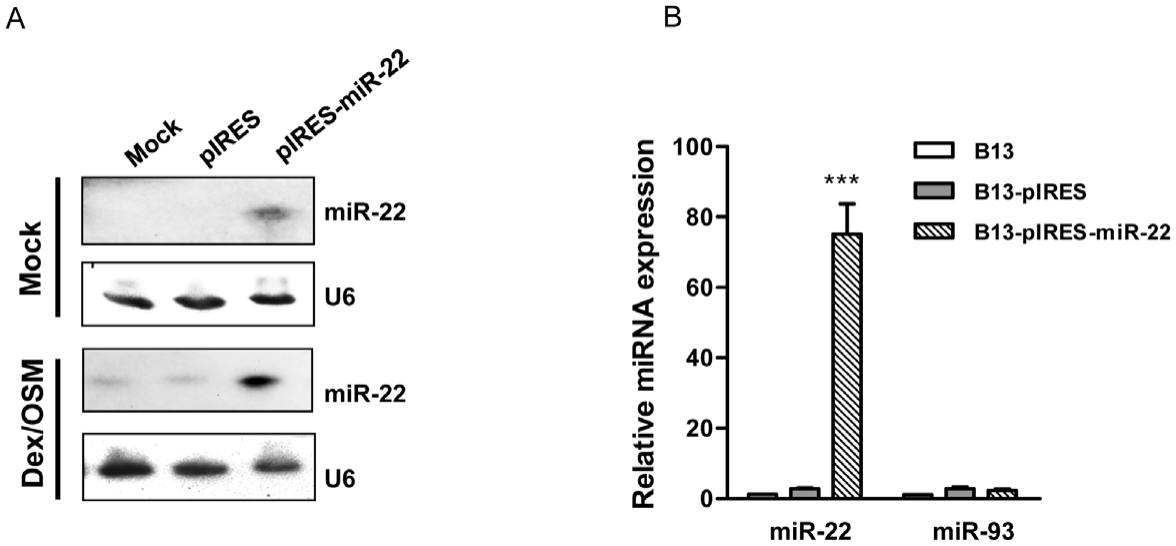
Ectopic overexpression of miR-22 in AR42J-B13 cells was analyzed by Northern blot (A) and stem-loop real-time PCR analysis (B). (A) pIRES is the empty vector control. pIRES-miR-22 is the miR-22 expression vector. (B) B13-pIRES is the AR42J-B13 cells stably transfected with the pIRES vector control, while B13-pIRES-miR-22 is the AR42J-B13 cells stably transfected with the miR-22 expression vector. MicroRNA-93 was used as a negative control. (***, p<0.001).

**Figure 4:**
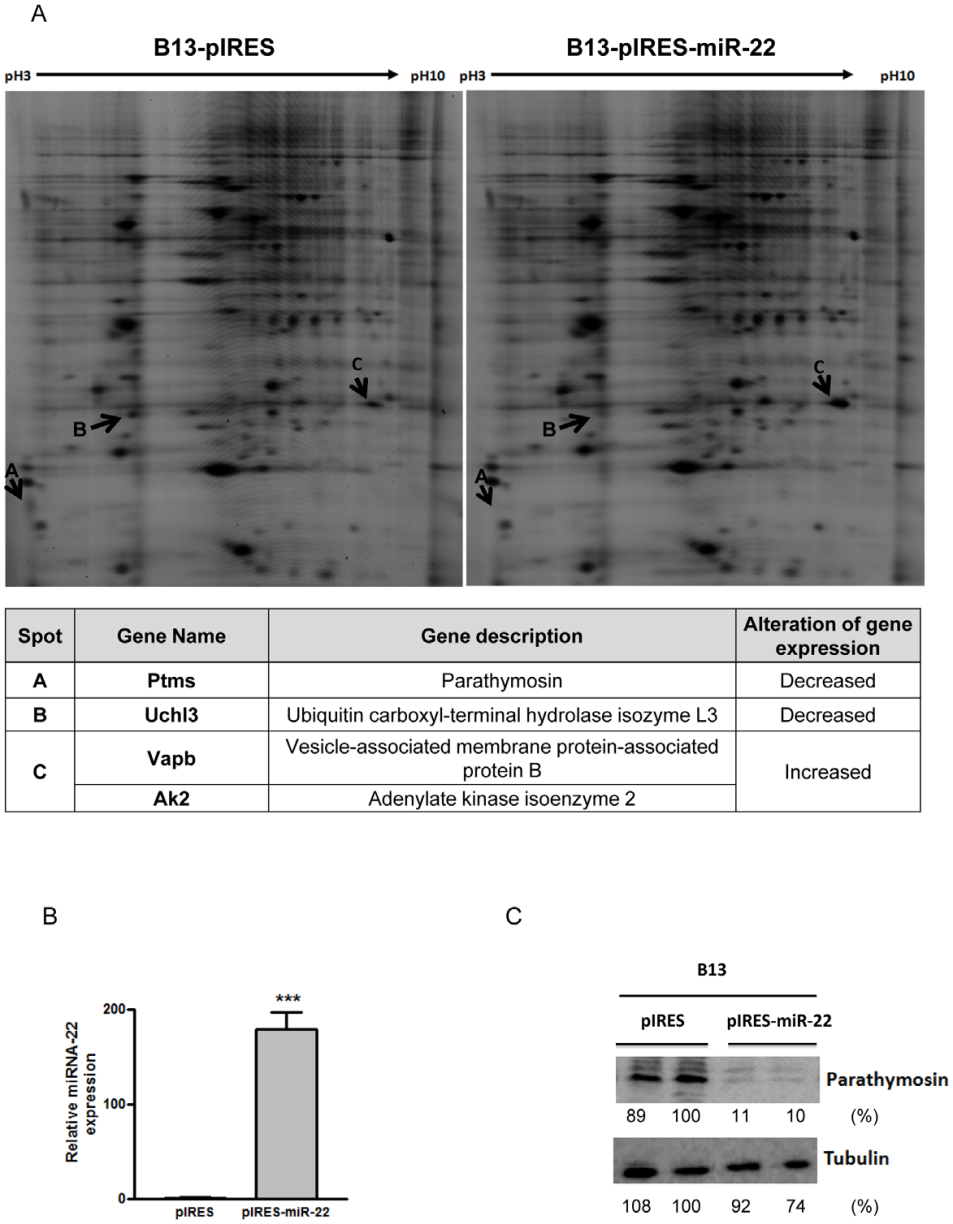
2D-DIGE analysis identified differentially expressed proteins in miR-22 overexpressing AR42J-B13 cells. Protein lysates of pooled B13-pIRES or B13-pIRES-miR-22 cells were labeled with Cy3 and Cy5, respectively (Experimental Procedures). Three differentially expressed features, Features A, B and C, were picked up for identification by LC-MS/MS. Proteins with altered expression were indicated by arrows. The candidate proteins identified were listed in the table below the image. Parathymosin protein was down-regulated as assayed by 2D-DIGE (A) and Western blot analysis (C) in AR42J-B13 cells stably transfected with pIRES-miR-22. The miR-22 expression level in transiently transfected AR42J-B13 cells was measured by stem-loop real-time PCR (B). (***, p<0.001).

To focus on the less well studied miR-22, we attempted to identify the potential targets of miR-22. We pooled AR42J-B13 cells stably transfected with pIRES2-EGFP-miR-22. The cloning and construction of this miR-22 expression vector is as described in the Experimental Procedures. Our miR-22 sequences are identical to those available in the miR database (Figure S2). These transfected cells were enriched by cell sorter on EGFP positive cells and followed by G418 selection. Mature miR-22 in these stably transfected cells were significantly higher than that of the vector control by Northern blot and real-time PCR (Fig. 3A and B). To identify the targets of miR-22, we first performed 2D-DIGE and LC/MS/MS analysis using these pooled cells stably overexpressing miR-22. Three differentially expressed features were subjected to LC/MS/MS analysis for protein identification. Four proteins with altered expression in miR-22 stably overexpressing cells were identified (Fig. 4A). Parathymosin (Ptms), and ubiquitin carboxyl-terminal hydrolase isozyme L3 (Uchl3) were reduced in expression, while vesicle-associated membrane-associated protein B (Vapb) and adenylate kinase isoenzyme 2 (AK2) were increased. To validate a target of miR-22, we chose to focus on parathymosin. Transient transfection of miR-22 into AR42J-B13 cells resulted in a high level of miR-22 by stem-loop RT-PCR (Fig. 4B), and approximately 10-fold reduction of parathymosin protein by Western blot analysis (Fig. 4C).

### Reduction of parathymosin mRNA and protein by treatments of Dex/OSM induction or miR-22 overexpression

**Figure 5:**
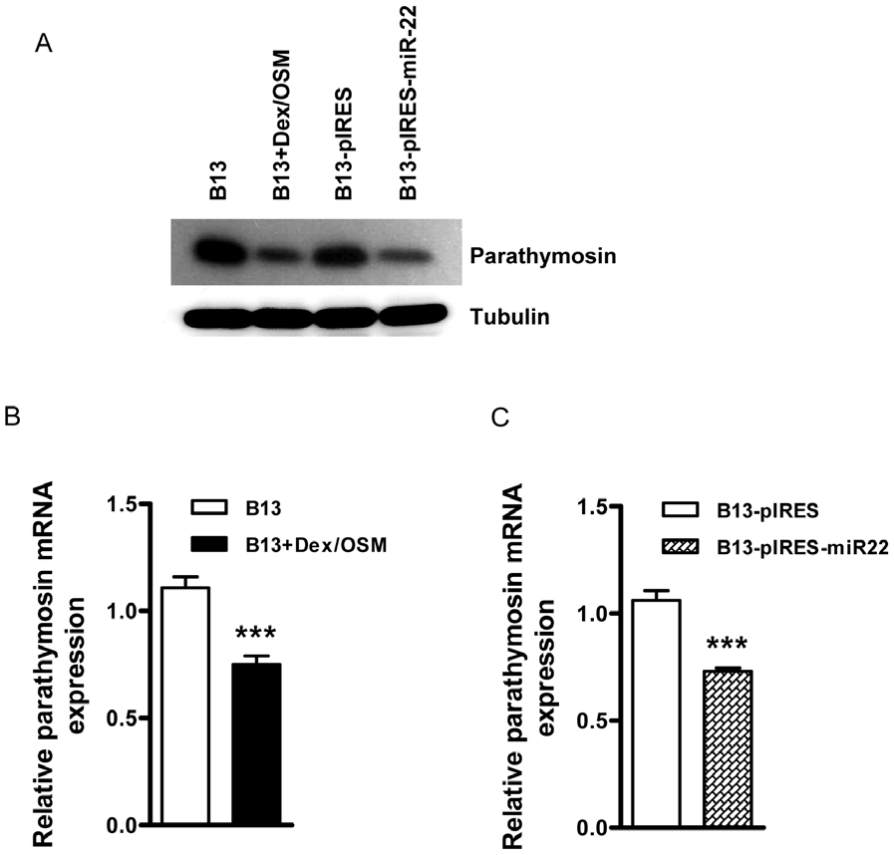
Reduction of parathymosin mRNA and protein levels were observed in AR42J-B13 cells treated with Dex/OSM or a miR-22 expression vector. (A) Reduced expression of parathymosin protein in AR42J-B13 cells was induced by Dex/OSM treatment (B13+DM) or by stable expression of miR-22 using Western blot analysis. (B & C) The expression of parathymosin mRNA was reduced to approximately 70% level in Dex/OSM induced AR42J-B13 cells (B), and in the stably transfected AR42J-B13 cells overexpressing miR-22 (C). The mRNA level was measured by real-time PCR analysis (***, p<0.001).

To confirm the results from proteomic analysis in Fig. 4A, we performed Western blot analysis using anti-parathymosin antibody and whole cell lysates from AR42J-B13 cells, with or without Dex/OSM induction, as well as with or without stable transfection using a pIRES-miR-22 expression vector (Fig. 5A). A similar degree of reduction of parathymosin protein in AR42J-B13 cells was detected by comparing Dex/OSM induction with miR-22 overexpression side-by-side. In addition to the parathymosin protein level, we also measured the parathymosin mRNA levels of AR42J-B13 cells treated with Dex/OSM or with miR-22 overexpression, by real time PCR analysis (Fig. 5B and 5C). In both cases, the mRNA levels of treated samples were reduced to approximately 70% of the untreated controls.

### Bioinformatic analysis of the 3′ UTR of parathymosin mRNA

**Figure 6:**
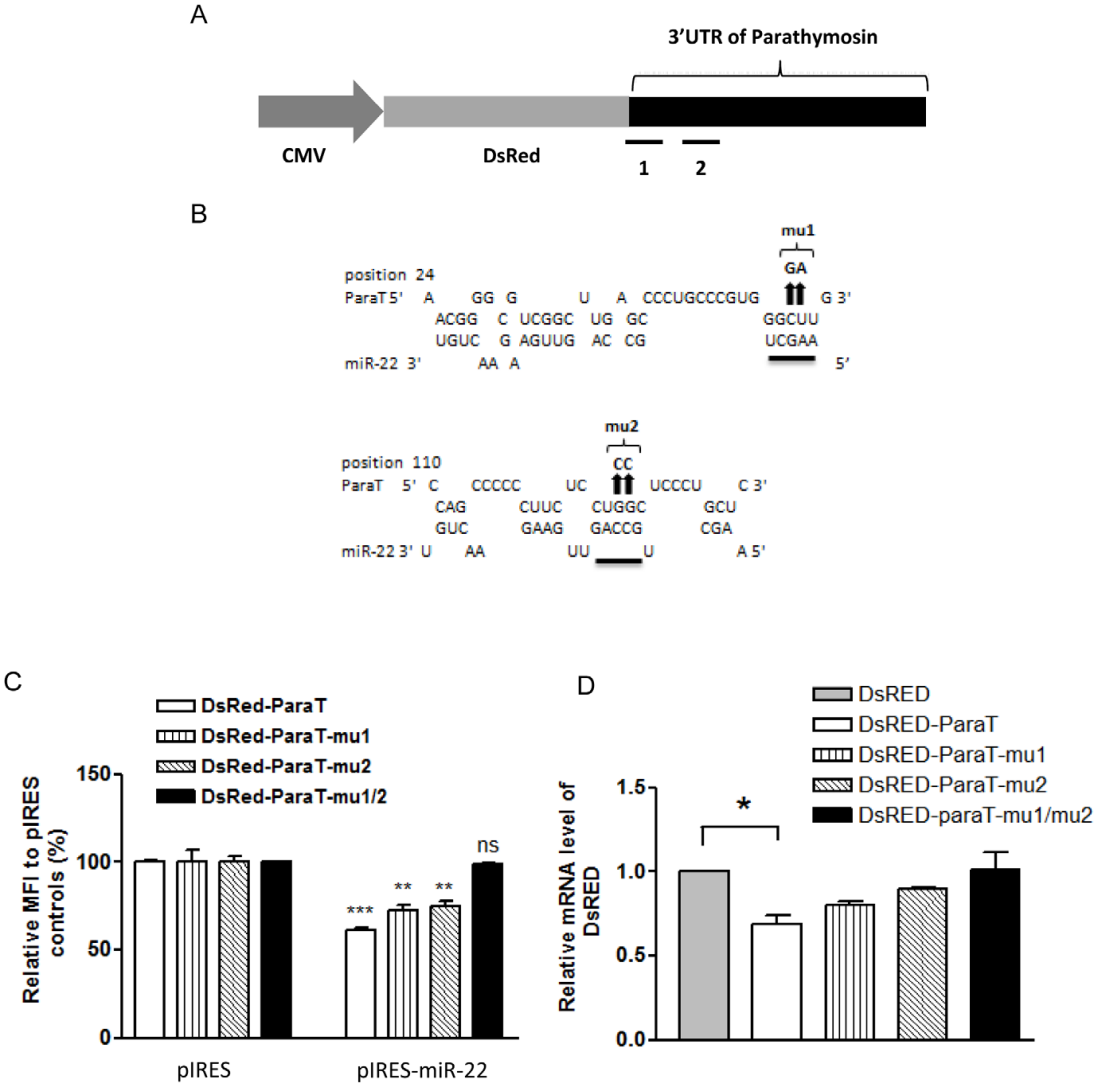
The suppression effect of miR-22 on the expression of parathymosin depends on the predicted miR-22 target sites at the 3′UTR of parathymosin. (A) RNA Hybrid software predicted two miR-22 binding sites, designated as “1” and “2” in the cartoon, at the 3′UTR of parathymosin with free energy less than −20 kcal/mol. The reporter plasmid was engineered by fusing DsRED with the rat parathymosin 3′UTR. (B) Site-directed mutagenesis at the predicted miR-22 binding sites of the parathymosin 3′UTR were illustrated. Two nucleotide substitutions were introduced into each of the two predicted binding sites. The arrows indicate the mutation sites at the 3′UTR of parathymosin. The sequences at the 3′UTR of parathymosin were changed from 5′-CU-3′ to 5′-GA-3′ in mu1, and from 5′-GG-3′ to 5′-CC-3′ in mu2. (C) The reporter assay was performed by transient cotransfection of AR42J-B13 cells with the plasmid of a DsRED reporter containing various wild type and mutant parathymosin 3′UTR, and plasmid pIRES-miR-22. The mean fluorescence intensity (MFI) of DsRED was measured by FACS analysis gated on DsRED positive cells. The relative MFI was calculated by normalization to the pIRES control. Mutations at both predicted sites have an additive effect on miR-22 mediated silencing (***, p<0.001; **, p<0.005; ns, not significant). (D) The reduction of the DsRED reporter mRNA, containing wild type parathymosin 3′UTR, was abolished by mutations at the predicted binding sites of miR-22. Real-time PCR analysis on DsRED mRNA was performed by using the same reporter assay as described above in Fig. 6C. (*, *p*<0.5).

The 3′UTR region of parathymosin is highly conserved across human mouse and rat (Figure S3). The reduction of parathymosin could be caused by a direct or indirect effect from miR-22. In the former case, miR-22 could directly bind to the 3′ UTR of parathymosin mRNA, and mediate its suppression of gene expression. In the latter case, miR-22 could mediate its effect by influencing other cellular genes which in turn reduced the expression of parathymosin. In theory, direct and indirect effects are not mutually exclusive. To distinguish between the potential direct and indirect effects from miR-22, we first conducted computer-aided analysis. Some target prediction algorithms, such as TargetScan 5.2 [21] and miRanda [22], predicted no miR-22 binding sites on the parathymosin 3′UTR with a free energy less than −20 kcal/mol (data not shown). It therefore appears that the effect of miR-22 on parathymosin could be mediated through an indirect mechanism. However, other software programs, such as RNA Hybrid [23], predicted two miR-22 binding sites at the 3′UTR of parathymosin (free energy less than −20 kcal/mol). These predicted target sites are highly conserved in human and rodents (Figure S3). As shown in Fig. 6A and 6B, there is no perfect match between the putative seed region of miR-22 and the two predicted target sites on parathymosin 3′UTR. These features suggest that the predicted target sites of miR-22 at the 3′UTR of parathymosin may not be convincing. On the other hand, it is well known in the literature that some of the socalled unconventional target sites for miRNA binding could involve some imperfect match of base pairing between the seed region and the target site [24]–[26].

### MiR-22 inhibited the expression of parathymosin through the 3′UTR binding sites

To experimentally test the possibility of any unconventional target sites of miR-22 on the 3′ UTR of parathymosin, we performed the reporter assay using a DsRed reporter containing the parathymosin 3′UTR. The relative mean fluorescence intensity (MFI) was calculated by FACS analysis. Comparing with the pIRES vector control, miR-22 inhibited the reporter expression by about 50%. We next performed site-directed mutagenesis at each predicted miR-22 target sites at the 3′ UTR of parathymosin. Two nucleotide changes were introduced into each predicted binding sites (Fig. 6B). The DsRed-parathymosin 3′ UTR reporter plasmid (DsRed-ParaT) was cotransfected with the pIRES vector control plasmid or pIRES-miR-22 expression plasmid into AR42J-B13 cells. As shown in Fig. 6C, the wild type reporter (DsRed-ParaT) activity was subjected to the suppression effect from pIRES-miR-22 (open bar). The effect appeared to be modest, yet highly reproducible. Mutations at a single miR-22 binding site (DsRed-ParaT-mu1 or DsRed-ParaT-mu2) did not abolish the suppression effect from pIRES-miR-22 on the reporter activity. However, mutations on both putative binding sites of the reporter plasmid (DsRed-ParaT-mu1/2) resulted in a reporter activity comparable to that of cotransfection with the pIRES control (solid bar) (Fig. 6C). The results of the reporter assay in Fig. 6C was confirmed by a real-time PCR assay for the mRNA level of DsRed reporter with either a wild type or a mutant parathymosin 3′UTR (Fig. 6D). The results indicated that miR-22 could reduce DsRed mRNA levels through its binding site on the parathymosin 3′UTR (Fig. 6D).

**Figure 7:**
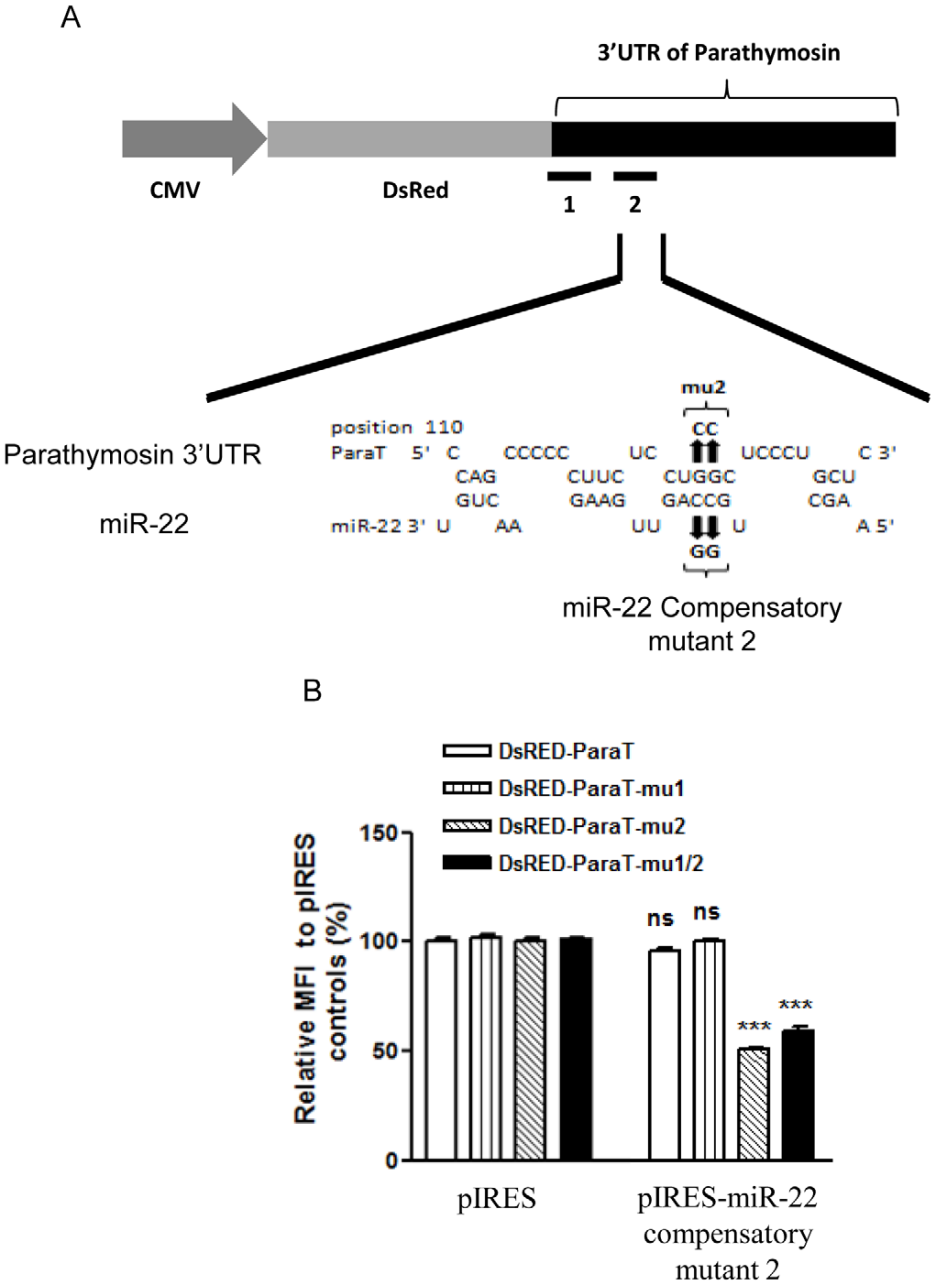
Compensatory mutations of miR-22 restored the repression effect of miR-22 on the 3′UTR of a parathymosin reporter containing a mu2 target site. (A) Compensatory mutation from 5′-CC-3′ to 5′-GG.-3′ at the predicted seed sequences of miR-22. (B) Reporter gene assay was performed by co-transfection into AR42J-B13 cells with the various DsRED reporter plasmids, containing various wild type and mutant parathymosin 3′UTR, and pIRES-miR-22 containing the compensatory mutation. FACS analysis was performed as in Fig. 6. (***, p<0.001; ns, not significant).

To further validate whether miR-22 can directly bind to the 3′UTR of parathymosin to suppress its expression, we generated compensatory mutations on the putative seed region of miR-22 that can restore the sequence complementarity to the mutated binding site 2 (Fig. 7A). Indeed, the pIRES-miR-22 compensatory mutant 2 restored the suppression effect on the reporter activity of 3′UTR mutant 2 and mutant 1/2 (Fig. 7B).

### Knockdown the endogenous miR-22 by LNA-based antagomiR-22

**Figure 8:**
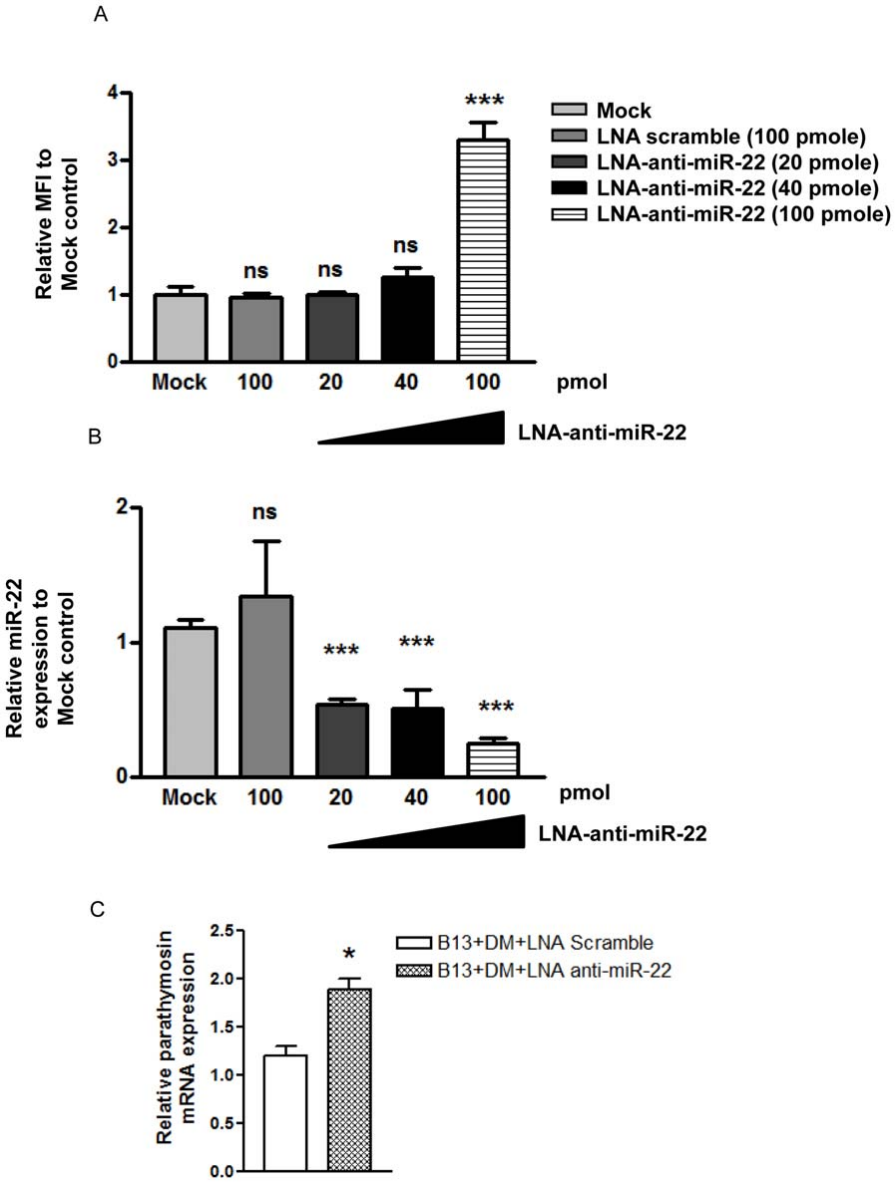
LNA-based antagomiR-22 increased parathymosin 3′UTR reporter activity in a hepatoma cell line Q7 by knockdown endogenous miR-22. (A) The reporter assay was conducted by co-transfection of DsRED-parathymosin 3′UTR with LNA-antagomiR-22 in Q7 cells using Lipofectamine 2000. Reporter activity was significantly increased when 100 pmol of LNA-anti-miR-22 was used. (B) Different concentrations of LNA-antagomiR-22 were used to knockdown the endogenous miR-22 expression level, which was measured by stem-loop real-time PCR. (***, p<0.001) (C) Parathymosin mRNA was increased significantly by stem-loop real-time PCR, when transdifferentiated AR42J-B13 cells were treated with LNA-anti-miR-22. (*, *p*<0.5).

Next, we asked whether knockdown of miR-22 could increase the reporter activity. We applied the locked nucleic acid (LNA) technology to deliver anti-miR-22 into a rat hepatoma cell line Q7 [5] to knockdown the endogenous level of miR-22. Consistent with results in Fig. 6 and 7, we observed the elevation of the cotransfected DsRed reporter activity (Fig. 8A). The efficacy of LNA anti-miR-22 was evaluated in Q7 cells by real-time PCR analysis of the miR-22 RNA level (Fig. 8B). Similarly, LNA knockdown of miR-22 in transdifferentiated AR42J-B13 cells resulted in a significantly increased level of parathymosin mRNA (Fig. 8C).

### An in vivo inverse correlation between miR-22 expression and the parathymosin mRNA level

**Figure 9:**
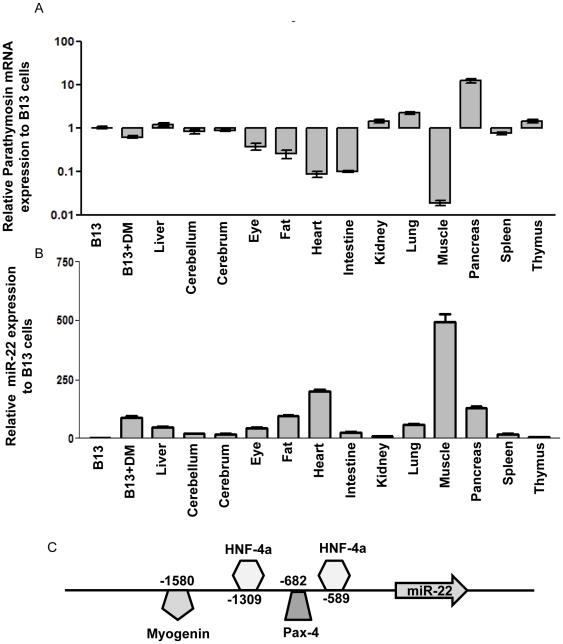
An inverse correlation between the expression levels of parathymosin mRNA and miR-22 was observed in most rat primary tissues. Real-time PCR quantified parathymosin mRNA expression profile in various rat primary tissues, including cerebellum, cerebrum, eye, fat, muscle, pancreas, kidney, heart, lung, spleen, thymus and liver. AR42J-B13 cells with or without Dex/OSM induced transdifferentiation were included as references. The relative mRNA level of parathymosin was normalized to that of untreated AR42J-B13 cells (A). The expression of miR-22 in these tissues and cells were measured by stem-loop real-time PCR (B). The binding sites of transcription factors on the miR-22 promoter are predicted by the Transfac software (27) and presented in the schematic illustration (C).

Consistent with the results in Fig. 6, 7, 8, we found an inverse correlation between parathymosin mRNA and miR-22 levels across different primary tissues of a female rat by real-time RT-PCR and stem-loop PCR analyses (Fig. 9A and 9B). For example, miR-22 is most abundant in muscle and heart, while parathymosin is the least abundant in muscle and heart. However, such an inverse correlation does not always hold true (e.g., pancreas). As shown in Fig. 9C, computational analysis of the promoter region of miR-22 predicts several binding sites for transcription factors, including HNF-4a, myogenin, and pax-4. Therefore, the high abundance of miR-22 in liver, muscle, and heart is most likely driven by these tissue specific transcription factors [8], [27]. Indeed, we found both pri-miR-22 and mature miR-22 were increased in expression, which correlated with HNF-4a mRNA level during AR42J-B13 transdifferentiation (Figure S4A). Pax-4 is a negative transcription factor which is transiently expressed during the embryonic development of pancreas [28]. Pax4 is not expressed in un-induced AR42J-B13 cells and only slightly induced after transdifferentiation (Figure S4B).

### MiR-22 is neither necessary nor sufficient for transdifferentiation

**Figure 10:**
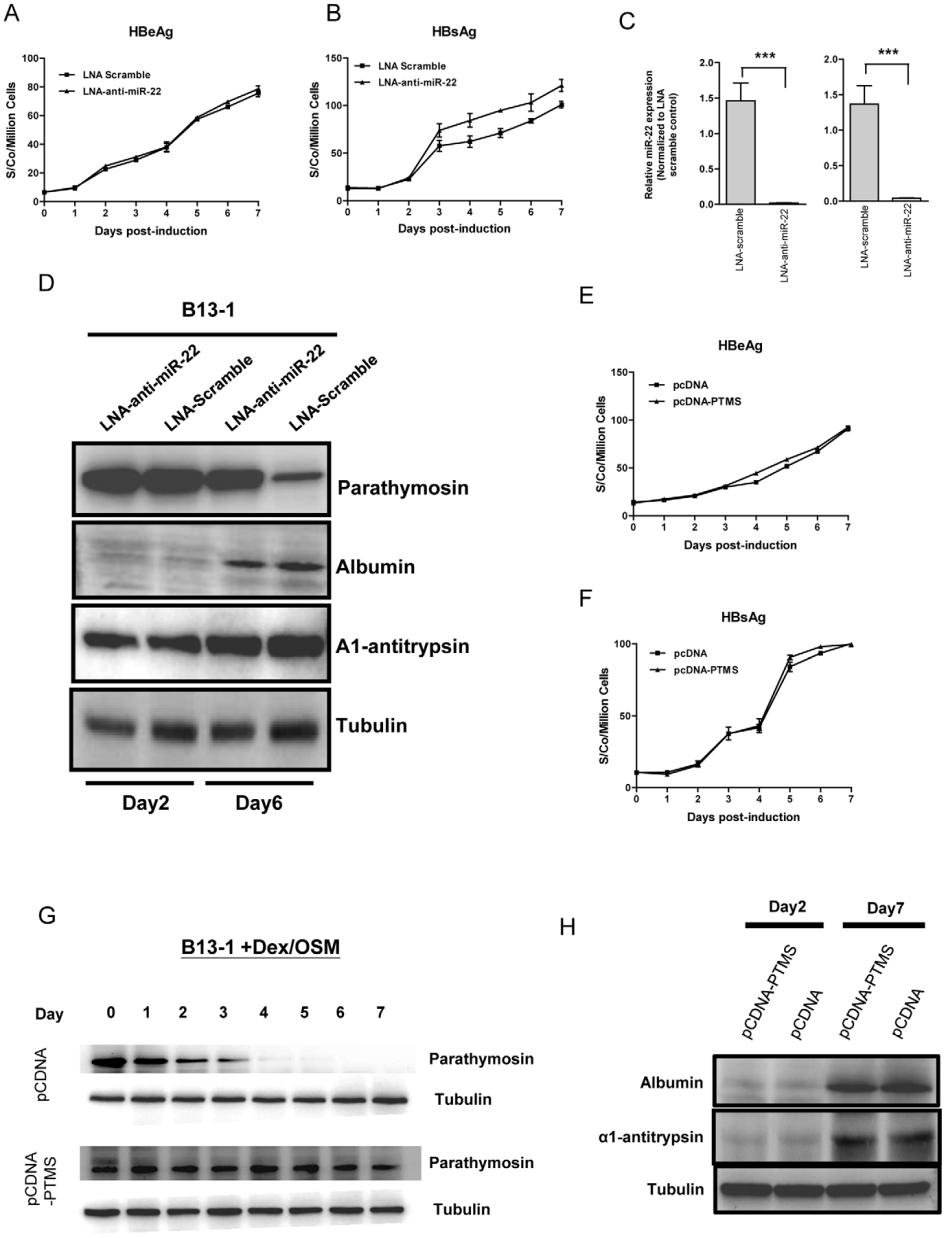
The potential effect of parathymosin or miR22 on the transdifferentiation of AR42J-B13 cells. (A) Similar levels of HBV e antigen (HBeAg) were detected in the media of B13-1 cells transfected with LNA anti-miR22 vs. a LNA scramble control. (B) Increased secretion of HBV surface antigen (HBsAg) was detected by ELISA in the medium of B13-1 cells transfected with LNA anti-miR22. (C) The reduction of the endogenous level of miR-22 in B13-1 cells after LNA anti-miR-22 treatment, was measured by real time PCR. (D) Treatment of B13-1 cells with LNA anti-miR22 resulted in no significant effect on transdifferentiation markers of alpha1-antitrypsin and albumin by Western blot analysis. (E & F) No significant effect on secreted HBsAg and HBeAg was detected by ELISA in B13-1 cells transfected with a parathymosin (PTMS) expression vector. (G) A time course of the gradual decrease of parathymosin protein in B13-1 cells was conducted by Western blot analysis. B13-1 cells were transfected with a vector control (*upper panel*) or a parathymosin expression vector (*lower panel*) prior to Dex/OSM induction. (H) Transdifferentiation markers of albumin and alpha1-antitrypsin were measured by Western blot analysis, using B13-1 cells transfected with a parathymosin expression vector or a control vector pCDNA prior to Dex/OSM induction.

As shown in Table 1 and 2, expression of many microRNAs changed upon transdifferentiation. It is also well known that each microRNA has a potential to target hundreds of genes [6]. Therefore, it is difficult to predict which microRNA species or which target genes might play a regulatory role in programming transdifferentiation. As a preliminary attempt to address this issue, we asked whether transdifferentiation can be perturbed by knocking down the endogenous miR-22 (Fig. 10A–10D) or by overexpressing parathymosin (Fig. 10E–10H). We introduced LNA-anti-miR-22 into a B13-1 cell line and monitored the expression of transdifferentiation markers in the transfected culture of B13-1 cells. This B13-1 cell line was established previously by stable transfection of AR42J-B13 cells with an HBV genome [4]. HBV-encoded surface antigen (HBsAg) and e antigen (HBeAg) can be released into the medium upon transdifferentiation. Using HBsAg and HBeAg as surrogate markers of transdifferentiation, we observed no difference in HBeAg secretion, when B13-1 cells were transfected with anti-miR-22 before Dex/OSM treatment (Fig. 10A; Experimental Procedures). In contrast, we observed a reproducible difference in the secreted amount of HBsAg by ELISA analysis (Fig. 10B). To further investigate this apparent contradiction between HBsAg and HBeAg, we examined other transdifferentiation markers, including albumin and alpha1-anti-trypsin (Fig. 10D), as well as HNF4a and C/EBP-alpha (data not shown), by Western blot analysis. Our studies suggest that the reduction of endogenous miR-22 has no significant effect on transdifferentiation in general, albeit a minor effect on HBsAg was noted (Fig. 10A; see Discussion).

Similarly, we asked whether parathymosin has any effect on hepatic transdifferentiation of B13-1 cells. As shown in Fig. 10E–10H, we detected no apparent effect on the expression of both viral and cellular transdifferentiation markers, when B13-1 cells were transfected with a parathymosin expression vector before Dex/OSM treatment (Material and Methods). The daily expression of the exogenous parathymosin protein was monitored on day 1 to day 7 post-induction by Western blot analysis (Fig. 10G).

As shown in Fig. 10, we have demonstrated that reduction of miR-22 or expression of parathymosin has no major effect on Dex/OSM-induced transdifferentiation. While this result strongly suggests that miR-22 is not *necessary* for transdifferentiation, it remains unclear if miR-22 expression could be *sufficient* for transdifferentiation. To address this issue, we cotransfected AR42J-B13 cells with a HBV replicon and pIRES-miR-22 without treatment of Dex/OSM, and detected no HBsAg in the medium by ELISA (data not shown). The same negative result in HBsAg ELISA was obtained when the same cotransfection experiment was performed by using B13-1 cells without Dex/OSM (data not shown). Taken together, miR-22 does not appear to be either necessary or sufficient for transdifferentiation of AR42J-B13 cells.

### MiR-22 can target parathymosin in human hepatocytes

**Figure 11:**
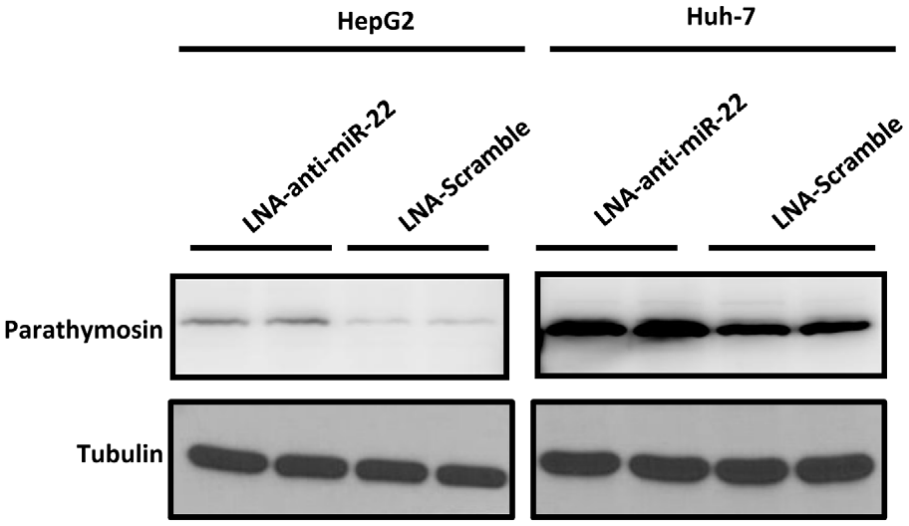
Parathymosin can be targeted by miR-22 in human hepatoma cell lines Huh7 and HepG2. Increased expression of parathymosin protein can be detected by Western blot when Huh7 and HepG2 cells were treated with LNA anti-miR-22 (Experimental Procedures).

So far, our studies on miR-22 and parathymosin have been based on the rat cells, such as Q7, AR42J-B13 and its derived B13-1 cells. To demonstrate the generality of our studies, we extended our research to human hepatoma cell lines Huh7 and HepG2. As shown in Fig. 11, we examined by Western blot analysis the expression of parathymosin in HepG2 and Huh7 cells transfected with LNA-anti-miR-22 or LNA-scramble control, respectively. Indeed, treatment with anti-miR-22 can result in elevated expression of parathymosin protein in both HepG2 and Huh7 cells.

In summary, we used miR-22 as a model system to examine the control of gene expression during hepatic transdifferentiation of AR42J-B13 cells.

## Discussion

We profiled the microRNA expression of rat AR42J-B13 cells before and after transdifferentiation into hepatocytes (Table 1 and 2). The expression of miR-22 was increased by more than 100-fold after hepatic transdifferentiation, as measured by real-time PCR analysis (Fig. 1B). To date, unlike miR-122a, miR-22 in hepatocytes has been less well studied [21], [29]. To understand better the biology of miR-22, we identified parathymosin as a potential target of miR-22 (Fig. 4A). We found that miR-22 could reduce the protein and mRNA expression of parathymosin (Fig. 4, 5, 6, 7, 8, 9, 10, 11). The biological significance of miR-22 and parathymosin is discussed below.

### Reduction of gene expression by miR-22

The reduction of the DsRed reporter mRNA in the transient transfection system (Fig. 6D) is consistent with the reduction of parathymosin mRNA in miR-22 overexpressing cell lines by real-time PCR analysis (Fig. 5C). These results were also confirmed by the microarray analysis (fold change 0.7) (data not shown). In addition to parathymosin, other cellular proteins, such as ubiquitin carboxyl-terminal hydrolase isozyme L3 (Uchl3), were also reduced in expression (Fig. 4A).

### Translational suppression vs. mRNA degradation

MicroRNAs can fine tune the gene expression by translational suppression or by promoting degradation of targeted mRNAs [30], [31]. As shown in Fig. 4C and Fig. 5A, stable transfection with pIRES-miR-22 can reduce the protein expression of parathymosin by 5–10 folds in Western blot analysis. However, the 5–10 fold effect of miR-22 on the protein expression of parathymosin (Fig. 4C) is much greater than its less than 2-fold effect on the mRNA level (Fig. 5C). Therefore, it is possible that miR-22 could affect both mRNA stability and protein translation of parathymosin through its direct targeting at the 3′UTR of parathymosin. Alternatively, miR-22 could have a highly significant indirect effect on the protein expression of parathymosin. For example, miR-22 could affect the expression of another gene(s) which in turn affects the expression of parathymosin.

### Elevation of gene expression by miR-22

As shown in Fig. 4A, the protein expression of vesicle-associated membrane-associated protein B (Vapb) and adenylate kinase isoenzyme 2 (AK2) were increased in cells overexpressing miR-22. It is well known that liver-enriched miR-122a can positively stimulate HCV replication in human hepatoma Huh7 cells. This positive effect is mediated through direct binding of miR-122a to two different target sites near the 5′ end of HCV RNA genome [32]. The detailed mechanism of the increased protein expression of Vapb and AK2 by miR-22, directly or indirectly, remains to be investigated in the future.

### Solo vs. clustering miRNA

The pattern of coordinated reduction in expression of miR-25, miR-93, and miR-106b (Table 1) is due to the clustering of these three miRNA genes at intron 12 of Mcm7 on chromosome 12. As such, they share the same promoter and are processed from the same precursor mRNA of Mcm7 [33], [34]. Similarly, genes encoding for miR-192 and miR-194 are clustered on chromosome 1, and could explain their coordinated increase in expression (Table 2). The genes encoding for miR-17-1/miR-17-5p, miR-18a, miR-19a, miR-20a, miR-19b-1, and miR-92a-1 are clustered on chromosome 15 [35]. While the reduction in expression of miR-17-5p, miR-18a, miR-20a, and miR-92 were well coordinated in transdifferentiation, the expression of miR-19a was not concordant with its neighboring microRNA genes. In contrast to the clustering miRNA genes as mentioned above, some solo microRNAs, such as miR-22 on chromosome 10 [35] and miR-122a on chromosome 18, can be expressed independently from the other miRNAs.

### Transcription factors and biogenesis of miR-22

As shown in Fig. 9C, the upstream sequences from the miR-22 gene contain two HNF-4a, one Pax-4, and one myogenin binding motifs. Presumably, the expression of miR-22 is driven by HNF-4a, which is highly abundant in transdifferentiated AR42J-B13 cells [3], [4]. As shown in Table 2, we found increased expression of liver specific miRNAs in transdifferentiated hepatocytes, including miR-122a, miR-21, miR-22, miR-182, miR-29 and miR-30. By computer software prediction, HNF-4 binding sites are also present in the 5′ upstream regions of miR-21, miR-30b and miR-30d. Similarly, the binding site of a liver specific transcription factor HNF-1 can be predicted in the 5′ upstream region of miR-122a, miR-192 and miR-194-2 [9], [27], [36]. It is worth mentioning here that the expression kinetics of primary miR-22 RNA precursor paralleled to that of mature miR-22, suggesting the lack of control at the level of posttranscriptional processing (Figure S4). Taken together, we speculate that these upregulated expressions of miRNAs in transdifferentiated hepatocytes are mainly orchestrated by liver specific transcription factors, such as HNF-1 and HNF-4a.

### Parathymosin biology

Parathymosin is a highly conserved small acidic nuclear protein. It is widely distributed in mammalian tissues (Fig. 9;[19], [37]). It can bind to glucocorticoid receptor as a coactivator [18], [19]. In addition, it can also affect the binding of histone H1 and nucleosome assembly. In this regard, parathymosin may be involved in chromatin remodeling and thus can regulate gene expression epigenetically [18]. Although parathymosin is predominantly localized in the nucleus, some studies also reported that it can be shuttling between nucleus and cytoplasm depending on the cell density and differentiation status [38]. Transdifferentiation of AR42J-B13 cells can be induced by Dex treatment which is a glucocorticoid hormone. Therefore, we speculate the possibility of a negative feedback loop to prevent over-induction of GR responsive genes.

### miR-22 is neither necessary nor sufficient for transdifferentiation

MiR-22 is not likely to be required for Dex/OSM induced transdifferentiation, since we observed no apparent effect on several transdifferentiation markers by transfecting LNA-anti-miR22 or by overexpressing parathymosin in B13-1 cells (Fig. 10). Nor is miR-22 sufficient for transdifferentiation in the absence of Dex/OSM, as we observed no detectable HBsAg when HBV was cotransfected with the miR-22 expresssion vector into AR42J-B13 cells (data not shown). Intriguingly, while we observed no significant effect on most transdifferentiation markers by reducing the endogenous miR-22 (Fig. 10B, 10C, and 10D), we detected small, yet reproducible, increase of HBsAg by ELISA after anti-miR22 treatment (Fig. 10A). Further analysis by Southern blot revealed no apparent effect of miR-22 on HBV replication (data not shown).

### Downregulated microRNAs in transdifferentiated hepatocytes

It is known that Dex treatment can cause growth arrest which precedes transdifferentiation [3], [4]. As shown in Table 1, both miR-93 and miR-130b were reduced in transdifferentiated hepatocytes. Interestingly, Yeung et al. reported that miR-93 and miR-130b have a potential to target tumor suppressor protein p53-induced nuclear protein 1 (TP53INP1) in HTLV-1 infected/transformed cells [39]. TP53INP1 is a stress-induced protein and plays a role in controlling cellular proliferation and apoptosis [40]. Therefore, we speculate here that downregulation of miR-93 and miR-130b can result in elevated expression of tumor suppressor TP53INP1, which in turn can cause growth arrest of AR42J-B13 cells leading to transdifferentiation. Consistent with this speculation, our microarray data revealed that the cell cycle-related pathway is ranked the first pathway among all the downregulated genes, including cyclin A2 and cyclin B2, upon Dex/OSM treatment (unpublished results). Further investigation of microRNAs could help elucidate the mechanism of hepatic transdifferentiation.

## Materials and Methods

### Cell culture and transfection

AR42J-B13 cells [3] and human hepatoma cell lines Huh7 [41], HepG2 [42] were obtained from laboratories as cited here. These cell lines were maintained in Dulbecco's modified Eagle's medium (DMEM) (Invitrogen) containing 10% fetal bovine serum (Hyclone) at 37uC in the presence of 5% CO2. The Genejuice (Novagen) and lipofectamine2000 (Invitrogen) transfection procedure was according to manufacturer's instructions.

#### Induction of transdifferentiation

AR42J-B13 cells were cultured in DMEM (Dulbecco's modified Eagle's medium). Dexamethasone (Dex) and oncostatin M (OSM) were purchased from Sigma and R&D systems, respectively. To induce transdifferentiation, Dex and OSM were added at working concentrations 1 µM and 10 ng/ml, respectively. The medium of the cells treated with Dex/OSM were replenished every 2 days and maintained for 7 days before assay.

#### Rno-miR-22 cloning

The sequence of miR-22 was retrieved from Ensembl database and mirbase. The following primers were used to clone the full length stem-loop of miR-22: rno-miR-22-F:5′-TCAGCTGTCCTCTCCCTCAT-3′ and rno-miR-22-R:5′-AGTGTCCCCTTTCCCTGAGT-3′. The PCR product was sub-cloned from TA cloning vector (RBC) to pIRES2-EGFP (Clonetech) by EcoRI and BamH1 digestion. The forward and reversed clones were confirmed by sequencing.

#### Pooled stable miR-22 overexpressing cell lines

Approximately 1×10^6^ AR42J-B13 cells were transiently transfected by 3 µg plasmid DNA (pIRES2-EGFP-miR-22) with Genejuice (Novagen). The transfection efficiency was monitored by flowcytometry analysis. Following G418 selection for three weeks, the remaining colonies were pooled together and sorted again by FACS. Only EGFP positive cells were collected. These pooled stable cell lines were then maintained for 3 weeks before assay.

#### miRNA microarray (Ambion)

miRNAs were extracted by *mir*Vana™ miRNA Isolation Kit, and subsequently enriched by flashPAGE™ Fractionator. Fifty µg of total RNA were subject to electrophoresis in pre-cast gel and the RNA species less than 40 nt were eluted from the gel. The elution was further clean-up and concentrated by flashPAGE™ Reaction Clean-Up Kit. After spin-vac drying, the enriched small RNA was labeled with Ambion mirVana™ miRNA Labeling Kit and subject to microarray analysis. Small RNAs containing mature miRNAs were labeled with the mirVana™ miRNA labeling kit and hybridized to mirVana™ miRNA Bioarrays v.9.2 according to the vendor's protocol. Version 9.2 of mirVana microarray coincides with version 9.2 of miRBase sequence database [35], containing human (471 miRNAs), mouse (380 miRNAs) and rat (238 miRNAs) probes. Microarrays were scanned with a GenePix 4000B scanner (Axon instruments, GenePix software version 5.0). The data was imported into Genespring (Agilent) for normalization and clustering analysis.

#### Real-time PCR analysis

cDNA was synthesized by High Capacity cDNA Reverse Transcription kit (Applied Biosystem) according to manufacturer's manual. Briefly, 10 µg RNA was reverse transcribed into cDNA using random primers at 37°C for 120 minutes. The cDNA was then diluted 100 times for real-time PCR analysis using Power SYBR Green PCR master mix (Applied Biosystem). The real-time PCR was performed using default condition in 20 µl reactions by Applied Biosystems 7500 Real-Time PCR System and data were analyzed by relative quantification methods (ΔΔCt methods) using 7500 software V2.0.1.

#### Stem-looped miRNA real-time PCR

Taqman RT and stem-loop real-time assay were purchased from Applied Biosystems. miR-22 (assayID: 000398), miR-122a (assayID: 002245) and miR-93 (assayID: 001090) were used according to manufactory's manual. Briefly, 100 ng RNAs were reverse transcribed by specific stem-loop primer and further analyzed by Taqman assay real-time PCR using default setting. U6 snRNA (assayID: 001973) was used as an internal loading control. Data were analyzed by Applied Biosystems 7500 Real-Time PCR System and 7500 software V2.0.1.

#### miRNA Northern blot analysis

20 µg of total mRNA per sample extracted by Trizol (Invitrogen) were mixed with equal volume of denaturing gel loading buffer (which contains 95% formamide, 18 mM EDTA, 0.025% xylene cyanol, 0.025% bromophenol blue and 0.025% SDS). The RNA samples were denatured by heat at 95°C for 5 min and place on ice for at least 2 minutes prior to electrophoresis. After denaturation, the RNA was resolved by 15% denaturing polyacrylamide gel (with 8 M urea in Tris-borate buffer, which contains 90 mM Tris, 90 mM Boric acid and 2 mM Na_2_EDTA with pH 8.3 at 25°C) at 15 mA for approximately 1 hour. The gel was then transferred to nylon H-bond membrane by semi-dry apparatus (Bio-Rad) at 18 volt for 30 minutes. The membrane was cross-linked by Ultraviolet Crosslinkers (UVP) with 7500×100 uj/cm^2^ and then subjected to pre-hybridization in (6 X SSC, 10X Denhart's solution and 0.2%SDS with 100 µg of salmon sperm DNA). After pre-hybridization, the membrane was hybridized with isotope-labeled antisense miRNA probe (miRNA sequences were retrieved from Sanger mirbase v16.0) at 37°C for 16 hours. The membranes were washed at room temperature three times by 2xSSC with 0.5% SDS, and further washed at 42°C before autoradiogram.

#### Protein preparation for 2D-DIGE analysis

AR42J-B13 cells stably transfected with either a miR-22 expression vector or a vector only control were grown to 90% confluence in 10 cm dish. After washing twice with 1X phosphate buffered saline (PBS), cells were lysed by adding 500 µl lysis buffer (8 M urea, 4% CHAPS) to each dish. The cells were scraped off from culture dishes and transferred into eppendorf tubes, followed by three repeated sonications (10-sec each time with a 5-sec interval). The cell lysates were centrifuged at 13,000 rpm for 5 mins at 4°C and the supernatants were transferred to new eppendorf tubes. The protein concentrations were determined by BCA assay kit (Thermo Scientific).

A total of 380 µg of protein lysates were used for CyDye staining by the following procedures. First, the protein lysates were adjusted to pH 8.5 by 1 M HEPES buffer and later labeled by CyDye DIGE Fluor minimal dyes (GE) with the ratio of 8 pmole/µg of protein at 4°C in dark. Rehydration loading was applied for first dimension IEF for IPG strip (13 cm, pH 3–10, GE) under 30 volts, 12 hours. IEF condition was set as follows (Ettan IEFphor): 100 V for 30 mins, 250 V for 30 mins, 500 V for 30 mins, 1000 V for 30 mins, and 2000 V for 30 mins, 4000 V for 30 mins and with gradient ramping to 8000 V to reach 50000 Vhr. The second dimension was performed by GE SE600 system in 12% SDS-PAGE at 200 volts for 4.5 to 5 hours. The gels were further scanned by Typhoon using 2D-DIGE format (cy2:520 BP40; cy3:580 BP30 and cy5:670 BP 30). 2D-DIGE Images were analyzed by Decyder and the features were determined by default setting of the software. Spots of interest were picked up by automatic Ettan spot picker followed by manufacturer's manual.

#### In-gel digestion

Picked spots of interest were washed in 25 mM NH_4_HCO_3_/50% ACN and let stand at room temp for 15–30 min. After wash, the gels were soaked in 100% acetonitrile for 5 min and applied to spin-vac to completely dry. The dried gels were then digested at 37°C for 16–24 hours in 10 µg/ml sequencing grade trypsin (Promega) with 25 mM NH_4_HCO_3_ (pH 8.0). After digestion, the trypsin solution was transferred to a new tube and combined with gel extractions containing 25–50 µl of 50% ACN/5% TFA for 1 hour. The combined extracts were dried by spin-vacuum centrifugation and subject to LC/MS/MS analysis.

#### LC/MS/MS analysis

The peptide mixture was separated by using reverse-phase capillary liquid chromatography (Magic C18AQ, 75 mm 3 11 cm; Microm BioResources) at a flow rate of 200 nl/min. The eluent was directly analyzed with ion-trap mass spectrometry (Finnigan LCQ Deca XP; Thermo Scientific). A survey scan followed by 3 collision induced dissociation events was used. We performed the peptide identification using an in-house version of Mascot v. 2.2 (Matrix science, London, United Kingdom). The data sets were searched against Uni-Prot/Swiss-Prot (400771 sequences) using the following constraints: only tryptic peptides with up to two missed cleavage sites were allowed; ±2 Da mass tolerances for MS and ±0.8 Da mass tolerances for MS/MS fragment ions. Oxidation (M) and carboxyamidomethylation (C) were specified as variable modifications. Peptides were considered as being identified if their Mascot individual ion score was higher than 30 (*p*<0.01). The MS together with MS/MS spectra were searched using the MASCOT 2.2 search engine (Matrix Science), allowing one missed cleavage (trypsin). Precursor error tolerance and MS/MS fragment error tolerance were set to ±2 Da and ±0.8 Da. Only fully tryptic peptides and a MS/MS score above 30 were accepted for peptide identification. For protein identification by shotgun proteomics, methionine oxidation and cysteine carboxyamidomethylation were selected as variable modification. Proteins with a total ion score confidence interval percentage (CI %) above 99% were considered positive identifications.

#### LNA anti-miR-22 knockdown of HepG2, Huh7 and B13-1 cell lines

Cells were cultured in 6-well plates in DMEM (Dulbecco's modified Eagle's medium) to 70% confluence. One day after seeding, cells were transfected with puromycin resistamt plasmid (pTRE2pur) and LNA-scramble control or LNA anti-miR-22 (Locked Nucleic Acid, Exiqon), using Lipofectamine 2000 (Invitrogen), according to the manufacturer's instructions. Twelve hours post-transfection, untransfected cells were removed by puromycin treatment (2 µg/ml) for 2 days. Treated cells were further incubated in the presence of 0.5 µg/ml puromycin for another 2 days before harvesting for Western blot analysis. To induce transdifferentiation of the HBV-transfected B13-1 stable cell line [4], Dex/OSM were added as described earlier. The expression of HBsAg and HBeAg were measured by ELISA using HBsAg or HBeAg diagnostic kits (General Biologicals Cooporation, Taiwan) according to the vendor's protocols.

#### Antibodies

In general, primary antibodies were diluted 500–1,000 fold for Western blot experiments. These primary antibodies include rabbit polyclonal anti-albumin antibody (Santa Cruz), rabbit polyclonal anti-alpha1-antitrypsin antibody (Sigma), rabbit polyclonal anti-parathymosin antibody (Abcam) and mouse polyclonal anti-tubulin antibody (GeneTex, Taiwan). Secondary antibodies were diluted 5,000-fold for Western blot, including goat polyclonal anti-mouse horseradish peroxidase (HRP) (Sigma), goat polyclonal anti-rabbit- HRP (Santa Cruz).

#### Ethics Statement

All animal experiments were conducted under protocols approved by Academia Sinica Institutional Animal Care & Utilization Committee (ASIACUC). Research was conducted in compliance with the principles stated in the *Guide for the Care and Use of Laboratory Animals*, National Research Council, 1996. RNA samples extracted from rat tissues were a generous gift from Dr. Yi-Shuian Huang at Academia Sinica, Taiwan.

## Supporting Information

Figure S1 **Northern blot analysis of miRNA of AR42J-B13 (B13) and its HBV-producing stable clones, B13-1 and B13-28, during Dex/OSM induced transdifferentiation (4).** Mature miRNA of miR-93, miR-106b, miR-130b, miR-21, miR-22 and miR-182 were differentially expressed after transdifferentiation. U6 RNA was used as a loading control of RNA.
(TIF)

Figure S2 **Sequence alignment of cloned rat (rno) miR-22 of AR42J-B13 origin with the reference genome from Ensembl database (ENSRNOG00000035620).** The primer sequences for cloning and quantitative real-time PCR were listed. One single nucleotide polymorphism (SNP) with an A to G change is highlighted with an arrow, which is located outside the mature miR-22 sequences.
(TIF)

Figure S3 **Predicted binding sites of microRNA-22 are highly conserved at the 3′UTR of parathymosin from human, mouse and rat.** The sequences at the 3′ UTR of parathymosin were retrieved from Ensembl database and miR-22 binding site prediction was performed by RNA Hybrid software. The sequences of cloned 3′UTR of rat parathymosin are identical to that of the reference genome from Ensembl database (ENSRNOG00000016386).
(TIF)

Figure S4 **The expression levels of mature and primary miR-22 RNAs were correlated with that of HNF4a during hepatic transdifferentiation.** (A) At different time points after Dex/OSM treatment, mature miR-22 and primary miR-22 transcripts in transdifferentiating AR42J-B13 cells were measured by real-time RT-PCR, respectively. (B) The expression levels of HNF-4a, myogenin and pax-4 were measured by real-time PCR after Dex/OSM treatment in AR42J-B13 cells.
(TIF)

## References

[1] Rao MS, Dwivedi RS, Subbarao V, Usman MI, Scarpelli DG (1988). Almost total conversion of pancreas to liver in the adult rat: a reliable model to study transdifferentiation.. Biochem Biophys Res Commun.

[2] Tosh D, Shen CN, Alison MR, Sarraf CE, Slack JM (2007). Copper deprivation in rats induces islet hyperplasia and hepatic metaplasia in the pancreas.. Biol Cell.

[3] Shen CN, Slack JM, Tosh D (2000). Molecular basis of transdifferentiation of pancreas to liver.. Nat Cell Biol.

[4] Wang RY, Shen CN, Lin MH, Tosh D, Shih C (2005). Hepatocyte-like cells transdifferentiated from a pancreatic origin can support replication of hepatitis B virus.. J Virol.

[5] Shih CH, Li LS, Roychoudhury S, Ho MH (1989). In vitro propagation of human hepatitis B virus in a rat hepatoma cell line.. Proc Natl Acad Sci U S A.

[6] Bartel DP (2009). MicroRNAs: target recognition and regulatory functions.. Cell.

[7] Chen JF, Tao Y, Li J, Deng Z, Yan Z (2010). microRNA-1 and microRNA-206 regulate skeletal muscle satellite cell proliferation and differentiation by repressing Pax7.. J Cell Biol.

[8] Lagos-Quintana M, Rauhut R, Yalcin A, Meyer J, Lendeckel W (2002). Identification of tissue-specific microRNAs from mouse.. Curr Biol.

[9] Xu H, He JH, Xiao ZD, Zhang QQ, Chen YQ (2010). Liver-enriched transcription factors regulate microRNA-122 that targets CUTL1 during liver development.. Hepatology.

[10] Esau C, Davis S, Murray SF, Yu XX, Pandey SK (2006). miR-122 regulation of lipid metabolism revealed by in vivo antisense targeting.. Cell Metab.

[11] Krutzfeldt J, Rajewsky N, Braich R, Rajeev KG, Tuschl T (2005). Silencing of microRNAs in vivo with ‘antagomirs’.. Nature.

[12] Landgraf P, Rusu M, Sheridan R, Sewer A, Iovino N (2007). A mammalian microRNA expression atlas based on small RNA library sequencing.. Cell.

[13] Bar N, Dikstein R (2010). miR-22 forms a regulatory loop in PTEN/AKT pathway and modulates signaling kinetics.. PLoS One.

[14] Pandey DP, Picard D (2009). miR-22 inhibits estrogen signaling by directly targeting the estrogen receptor alpha mRNA.. Mol Cell Biol.

[15] Xiong J, Du Q, Liang Z (2010). Tumor-suppressive microRNA-22 inhibits the transcription of E-box-containing c-Myc target genes by silencing c-Myc binding protein.. Oncogene.

[16] Ting Y, Medina DJ, Strair RK, Schaar DG (2010). Differentiation-associated miR-22 represses Max expression and inhibits cell cycle progression.. Biochem Biophys Res Commun.

[17] Zhang J, Yang Y, Yang T, Liu Y, Li A (2010). microRNA-22, downregulated in hepatocellular carcinoma and correlated with prognosis, suppresses cell proliferation and tumourigenicity.. Br J Cancer.

[18] Martic G, Karetsou Z, Kefala K, Politou AS, Clapier CR (2005). Parathymosin affects the binding of linker histone H1 to nucleosomes and remodels chromatin structure.. J Biol Chem.

[19] Okamoto K, Isohashi F (2005). Macromolecular translocation inhibitor II (Zn(2+)-binding protein, parathymosin) interacts with the glucocorticoid receptor and enhances transcription in vivo.. J Biol Chem.

[20] Patel JB, Appaiah HN, Burnett RM, Bhat-Nakshatri P, Wang G (2011). Control of EVI-1 oncogene expression in metastatic breast cancer cells through microRNA miR-22.. Oncogene.

[21] Lewis AP, Jopling CL (2010). Regulation and biological function of the liver-specific miR-122.. Biochem Soc Trans.

[22] John B, Enright AJ, Aravin A, Tuschl T, Sander C (2004). Human MicroRNA targets.. PLoS Biol.

[23] Rehmsmeier M, Steffen P, Hochsmann M, Giegerich R (2004). Fast and effective prediction of microRNA/target duplexes.. RNA.

[24] Thomas M, Lieberman J, Lal A (2010). Desperately seeking microRNA targets.. Nat Struct Mol Biol.

[25] Meng F, Henson R, Wehbe-Janek H, Smith H, Ueno Y (2007). The MicroRNA let-7a modulates interleukin-6-dependent STAT-3 survival signaling in malignant human cholangiocytes.. J Biol Chem.

[26] Lal A, Navarro F, Maher CA, Maliszewski LE, Yan N (2009). miR-24 Inhibits cell proliferation by targeting E2F2, MYC, and other cell-cycle genes via binding to “seedless” 3′UTR microRNA recognition elements.. Mol Cell.

[27] Wingender E, Dietze P, Karas H, Knuppel R (1996). TRANSFAC: a database on transcription factors and their DNA binding sites.. Nucleic Acids Res.

[28] Smith SB, Ee HC, Conners JR, German MS (1999). Paired-homeodomain transcription factor PAX4 acts as a transcriptional repressor in early pancreatic development.. Mol Cell Biol.

[29] Girard M, Jacquemin E, Munnich A, Lyonnet S, Henrion-Caude A (2008). miR-122, a paradigm for the role of microRNAs in the liver.. J Hepatol.

[30] Behm-Ansmant I, Rehwinkel J, Doerks T, Stark A, Bork P (2006). mRNA degradation by miRNAs and GW182 requires both CCR4:NOT deadenylase and DCP1:DCP2 decapping complexes.. Genes Dev.

[31] Nilsen TW (2007). Mechanisms of microRNA-mediated gene regulation in animal cells.. Trends Genet.

[32] Jopling CL, Yi M, Lancaster AM, Lemon SM, Sarnow P (2005). Modulation of hepatitis C virus RNA abundance by a liver-specific MicroRNA.. Science.

[33] Ambs S, Prueitt RL, Yi M, Hudson RS, Howe TM (2008). Genomic profiling of microRNA and messenger RNA reveals deregulated microRNA expression in prostate cancer.. Cancer Res.

[34] Wang J, Xiang G, Mitchelson K, Zhou Y (2011). Microarray profiling of monocytic differentiation reveals miRNA-mRNA intrinsic correlation.. J Cell Biochem.

[35] Griffiths-Jones S, Saini HK, van Dongen S, Enright AJ (2008). miRBase: tools for microRNA genomics.. Nucleic Acids Res.

[36] Hino K, Tsuchiya K, Fukao T, Kiga K, Okamoto R (2008). Inducible expression of microRNA-194 is regulated by HNF-1alpha during intestinal epithelial cell differentiation.. RNA.

[37] Haritos AA, Salvin SB, Blacher R, Stein S, Horecker BL (1985). Parathymosin alpha: a peptide from rat tissues with structural homology to prothymosin alpha.. Proc Natl Acad Sci U S A.

[38] Brand IA, Heinickel A, Soling HD (1991). Localization of a 11.5 kDa Zn(2+)-binding protein (parathymosin) in different rat tissues. Cell type-specific distribution between cytosolic and nuclear compartment.. Eur J Cell Biol.

[39] Yeung ML, Yasunaga J, Bennasser Y, Dusetti N, Harris D (2008). Roles for microRNAs, miR-93 and miR-130b, and tumor protein 53-induced nuclear protein 1 tumor suppressor in cell growth dysregulation by human T-cell lymphotrophic virus 1.. Cancer Res.

[40] Savkovic V, Gaiser S, Iovanna JL, Bodeker H (2004). The stress response of the exocrine pancreas.. Dig Dis.

[41] Nakabayashi H, Taketa K, Miyano K, Yamane T, Sato J (1982). Growth of human hepatoma cells lines with differentiated functions in chemically defined medium.. Cancer Res.

[42] Aden DP, Fogel A, Plotkin S, Damjanov I, Knowles BB (1979). Controlled synthesis of HBsAg in a differentiated human liver carcinoma-derived cell line.. Nature.

