# Supplementary figures and images for: MicroRNA-22 Can Reduce Parathymosin Expression in Transdifferentiated Hepatocytes
Source: PLoS One. 2012 Apr 6;7(4):e34116. doi: 10.1371/journal.pone.0034116 (PMC3320904; doi:10.1371/journal.pone.0034116)

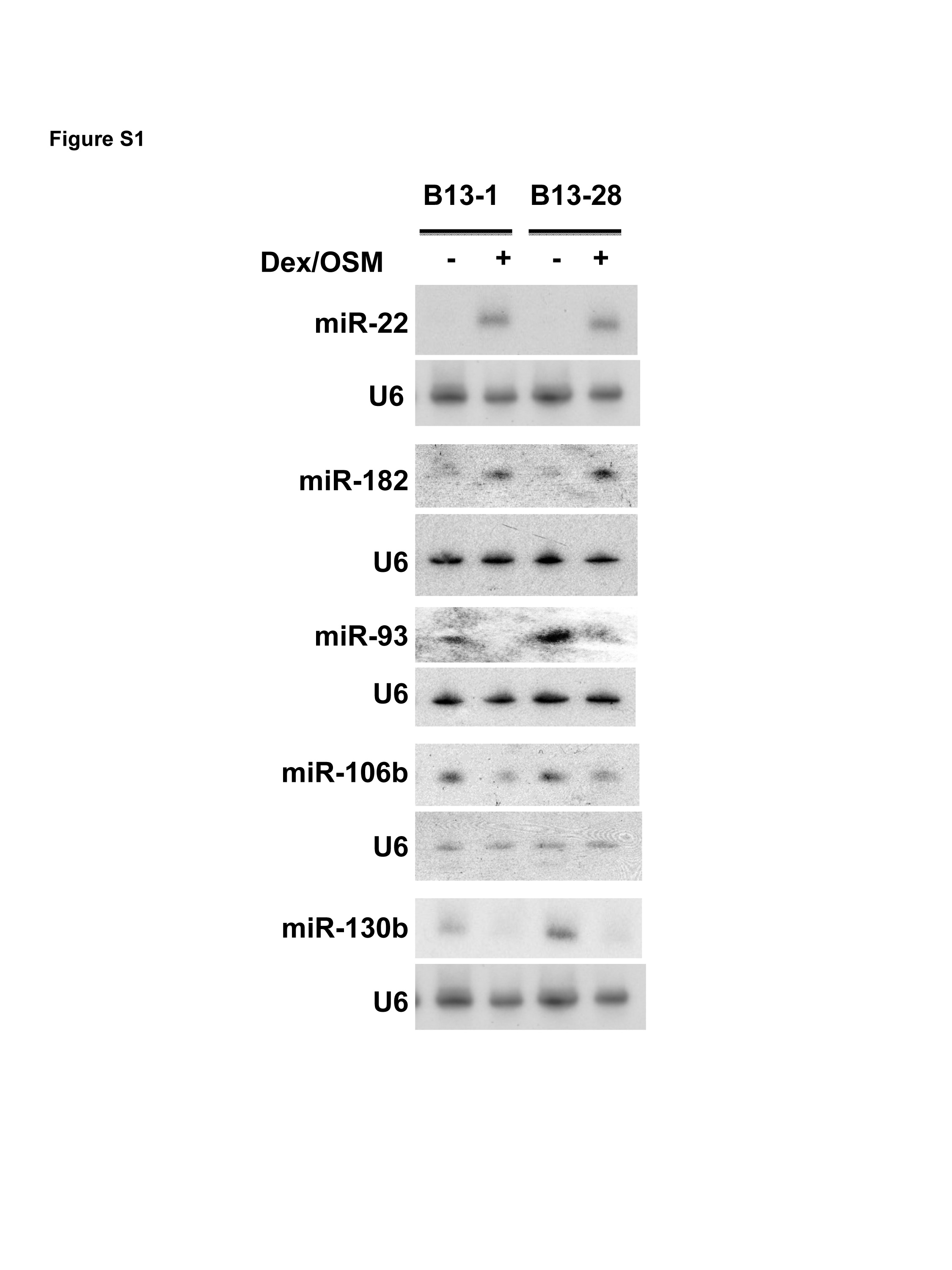

Supplement: Figure S1 — Northern blot analysis of miRNA of AR42J-B13 (B13) and its HBV-producing stable clones, B13-1 and B13-28, during Dex/OSM induced transdifferentiation (4). Mature miRNA of miR-93, miR-106b, miR-130b, miR-21, miR-22 and miR-182 were differentially expressed after transdifferentiation. U6 RNA was used as a loading control of RNA. (TIF) [file pone.0034116.s001.tif]

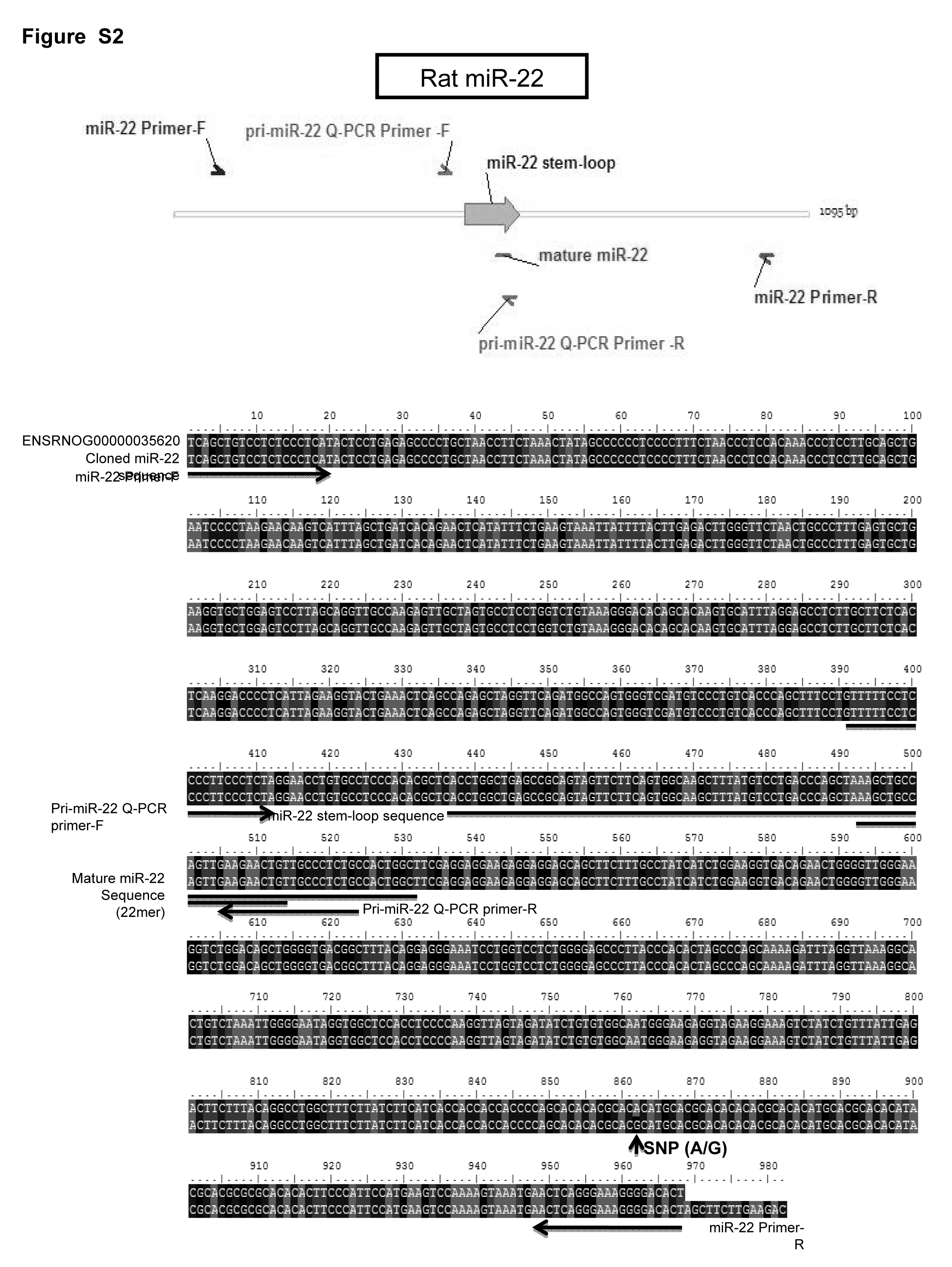

Supplement: Figure S2 — Sequence alignment of cloned rat (rno) miR-22 of AR42J-B13 origin with the reference genome from Ensembl database (ENSRNOG00000035620). The primer sequences for cloning and quantitative real-time PCR were listed. One single nucleotide polymorphism (SNP) with an A to G change is highlighted with an arrow, which is located outside the mature miR-22 sequences. (TIF) [file pone.0034116.s002.tif]

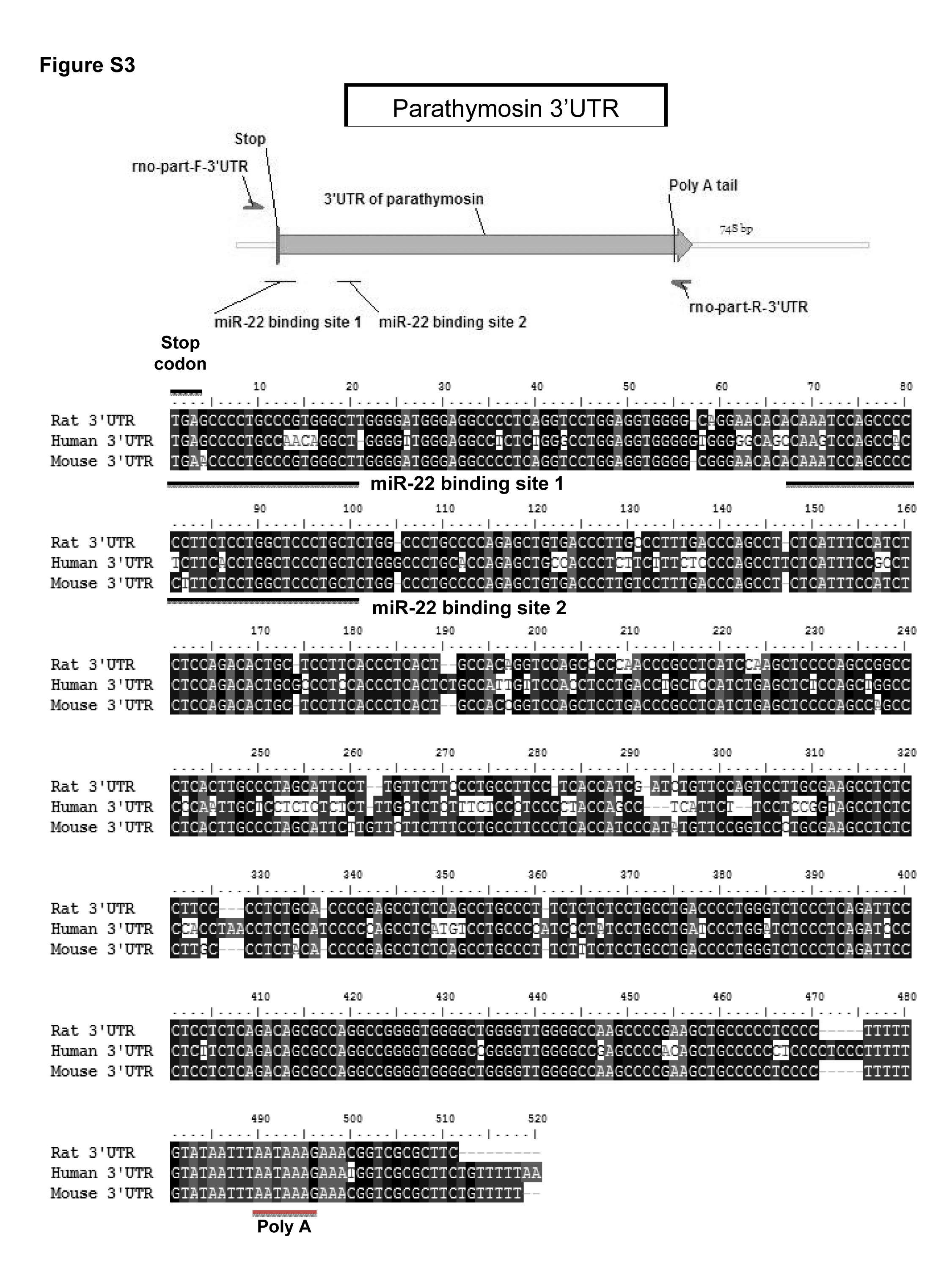

Supplement: Figure S3 — Predicted binding sites of microRNA-22 are highly conserved at the 3′UTR of parathymosin from human, mouse and rat. The sequences at the 3′ UTR of parathymosin were retrieved from Ensembl database and miR-22 binding site prediction was performed by RNA Hybrid software. The sequences of cloned 3′UTR of rat parathymosin are identical to that of the reference genome from Ensembl database (ENSRNOG00000016386). (TIF) [file pone.0034116.s003.tif]

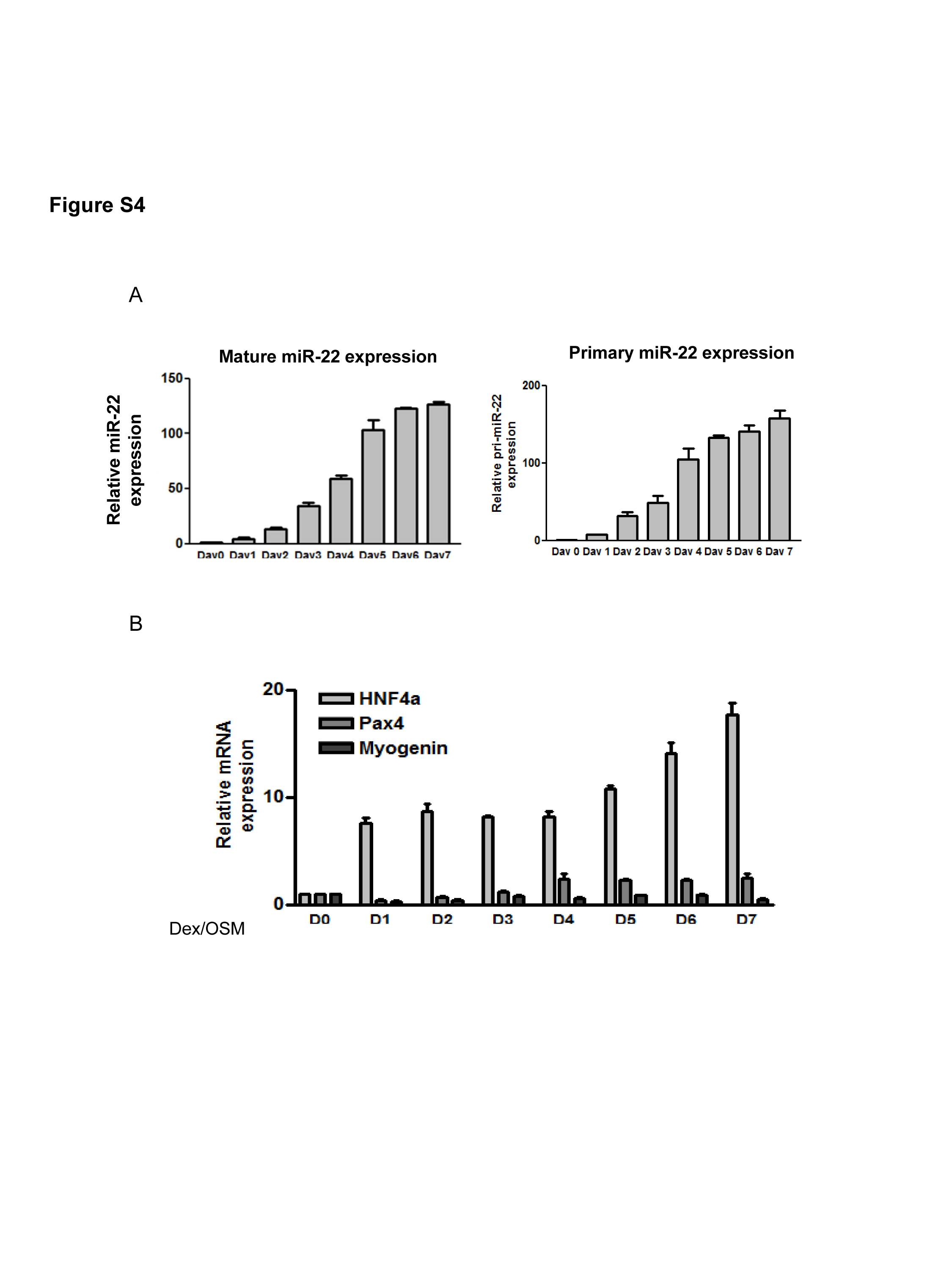

Supplement: Figure S4 — The expression levels of mature and primary miR-22 RNAs were correlated with that of HNF4a during hepatic transdifferentiation. (A) At different time points after Dex/OSM treatment, mature miR-22 and primary miR-22 transcripts in transdifferentiating AR42J-B13 cells were measured by real-time RT-PCR, respectively. (B) The expression levels of HNF-4a, myogenin and pax-4 were measured by real-time PCR after Dex/OSM treatment in AR42J-B13 cells. (TIF) [file pone.0034116.s004.tif]
